# Discovery of CD80 and CD86 as recent activation markers on regulatory T cells by protein-RNA single-cell analysis

**DOI:** 10.1186/s13073-020-00756-z

**Published:** 2020-06-24

**Authors:** Dominik Trzupek, Melanie Dunstan, Antony J. Cutler, Mercede Lee, Leila Godfrey, Lorna Jarvis, Daniel B. Rainbow, Dominik Aschenbrenner, Joanne L. Jones, Holm H. Uhlig, Linda S. Wicker, John A. Todd, Ricardo C. Ferreira

**Affiliations:** 1 Nuffield Department of Medicine, JDRF/Wellcome Diabetes and Inflammation Laboratory, Wellcome Centre for Human Genetics, NIHR Oxford Biomedical Research Centre, University of Oxford, Oxford, UK; 2 Department of Clinical Neurosciences, University of Cambridge, Cambridge, UK; 3 Translational Gastroenterology Unit and Department of Paediatrics, John Radcliffe Hospital, NIHR Oxford Biomedical Research Centre, University of Oxford, Oxford, UK

**Keywords:** Single-cell RNA sequencing (scRNA-seq), Multi-omics, CD4^+^ T cells, AbSeq, Immunophenotyping, CD80, CD86, Regulatory T cells (Tregs), C-C chemokine receptor type 9 (CCR9)

## Abstract

**Background:**

Traditionally, the transcriptomic and proteomic characterisation of CD4^+^ T cells at the single-cell level has been performed by two largely exclusive types of technologies: single-cell RNA sequencing (scRNA-seq) and antibody-based cytometry. Here, we present a multi-omics approach allowing the simultaneous targeted quantification of mRNA and protein expression in single cells and investigate its performance to dissect the heterogeneity of human immune cell populations.

**Methods:**

We have quantified the single-cell expression of 397 genes at the mRNA level and up to 68 proteins using oligo-conjugated antibodies (AbSeq) in 43,656 primary CD4^+^ T cells isolated from the blood and 31,907 CD45^+^ cells isolated from the blood and matched duodenal biopsies. We explored the sensitivity of this targeted scRNA-seq approach to dissect the heterogeneity of human immune cell populations and identify trajectories of functional T cell differentiation.

**Results:**

We provide a high-resolution map of human primary CD4^+^ T cells and identify precise trajectories of Th1, Th17 and regulatory T cell (Treg) differentiation in the blood and tissue. The sensitivity provided by this multi-omics approach identified the expression of the B7 molecules CD80 and CD86 on the surface of CD4^+^ Tregs, and we further demonstrated that B7 expression has the potential to identify recently activated T cells in circulation. Moreover, we identified a rare subset of CCR9^+^ T cells in the blood with tissue-homing properties and expression of several immune checkpoint molecules, suggestive of a regulatory function.

**Conclusions:**

The transcriptomic and proteomic hybrid technology described in this study provides a cost-effective solution to dissect the heterogeneity of immune cell populations at extremely high resolution. Unexpectedly, CD80 and CD86, normally expressed on antigen-presenting cells, were detected on a subset of activated Tregs, indicating a role for these co-stimulatory molecules in regulating the dynamics of CD4^+^ T cell responses.

## Background

Our understanding of the human immune system has been greatly influenced by the technological advances leading to the ability to precisely quantify mRNA and/or protein expression at the single-cell level. In particular, the implementation of flow cytometry as a routine and widely accessible research tool has shaped much of our current knowledge about the complexity of the immune system. With increased availability of fluorochrome-conjugated antibodies and more powerful lasers, flow cytometric assays allow typically 15–20 parameters that can be assessed in parallel. Developments in single-cell mass cytometry (CyTOF) have similarly allowed the simultaneous assessment of the expression of up to 50 protein targets using heavy metal-labelled antibodies [1].

The advent of single-cell RNA sequencing (scRNA-seq) has provided an unprecedented opportunity to investigate the global transcriptional profile at the single-cell level. In contrast to cytometry-based technologies, which are limited to the concurrent detection of up to a few tens of protein markers, scRNA-seq technologies allow to profile the entire transcriptome, with a recent explosion in different platforms becoming available to immunologists [2, 3]. These fundamentally differ in the cell capture methods and resulting sensitivity, ranging from a few hundreds of cells profiled with high sensitivity using plate-based capture methods such as SMART-seq2 [4], to tens of thousands of cells profiled with lower sensitivity using whole-transcriptome scRNA-seq platforms, such as 10× Genomics [5], Seq-Well [6] or Drop-seq [7].

Despite the growing popularity of whole-transcriptome scRNA-seq, two main issues still affect the performance of these platforms: cost and sensitivity. Even at high sequencing coverage, resulting in increased sequencing costs, stochastic dropout is a well-known problem in scRNA-seq, leading to an inflation of zero expression measurements. Furthermore, although several methods have been developed to impute missing expression values, questions remain about the performance of these methods [8]. This technical limitation is particularly relevant for resting primary cells, such as CD4^+^ T cells, and mainly limits the robust detection and quantification of lowly expressed genes, including lineage-defining transcription factors, which are critical for cell type identification and functional annotation. An important recent technical advance has been the development of new methods, such as CITE-seq [9] and REAP-seq [10], allowing the combination of whole-transcriptome scRNA-seq with measurement of protein expression at the single-cell level using oligo-conjugated antibodies. These methods provide increased clustering resolution and critical insight into the cell function, although the resulting sequencing cost, especially when combining large numbers of antibodies targeting highly expressed proteins, still limits the use of this technology as a widely applicable immunophenotyping tool.

In this study, we describe an integrated targeted scRNA-seq workflow, which we employ to simultaneously quantify the expression of 397 genes at the mRNA level and up to 68 genes at the protein level (using the Becton Dickinson (BD) AbSeq technology). We sought to assess the sensitivity and cost-effectiveness of this multi-omics system to immunophenotype human primary CD4^+^ T cells at the single-cell level and to identify discrete cell states providing potential new insight into the functional heterogeneity of T cells. By combining the expression of a targeted set of genes with the highly quantitative measurement of key protein markers, we have generated a high-resolution map of human CD4^+^ T cells in the blood and tissue and delineated distinct trajectories of T cell differentiation associated with a gradient of activation, apparent even in resting primary cells. Our data also showed very clearly the frequent low correlation between mRNA and protein expression in primary CD4^+^ T cells, thereby challenging the current view that our understanding of the cellular heterogeneity of the immune system can be re-defined based on single-cell transcriptional data alone. These attributes provided novel evidence for the expression of the B7 family molecules CD80 and CD86 on the surface of human primary Tregs ex vivo, thus revealing a biomarker for activated Tregs in circulation. We confirmed the upregulation of B7 molecules in CD4^+^ T cells activated in vitro and showed that IL-2 signalling was sufficient for the maintenance of B7 protein expression. Furthermore, we also identified a subset of CCR9^+^ effector T cells (Teffs) in circulation characterised by the expression of homing receptors and immune checkpoint molecules such as ICOS, CTLA-4, TIGIT, LAG-3 and TIM-3. These data provide insight into the function and specific surface markers that could be used to monitor the frequency of this rare CCR9^+^ T cell subset in the context of gut inflammatory diseases.

## Methods

### Subjects

Study participants included one systemic lupus erythematosus (SLE) patient (37-year-old female), recruited from the Cambridge BioResource, and one T1D patient (16-year-old male) and one autoantibody-negative healthy donor (14-year-old male) recruited from the JDRF Diabetes–Genes, Autoimmunity and Prevention (D-GAP) study.

Characterisation of total CD45^+^ immune cells isolated from a paired blood and duodenal biopsy was performed in cells isolated from two paediatric coeliac disease (CD) patients with active disease (one 5-year-old male with Marsh scale disease score of 3c and one 15-year-old male with Marsh scale disease score of 3b).

Flow cytometric assessment of the expression of CD80 and CD86 in CD4^+^ T cells was performed in four adult healthy donors (two females 46 and 67 years old and two males 41 and 51 years old), recruited from the Oxford Biobank and Cambridge BioResource. Treg in vitro expansion assays were performed in three adult healthy volunteers recruited via the CAMSAFE study either locally or from NHS Blood and Transplant (Cambridge).

### Cell preparation and FACS sorting

T cell assays were performed on cryopreserved peripheral blood mononuclear cells (PBMCs). Cryopreserved PBMCs were thawed at 37 °C and resuspended drop-by-drop in X-VIVO15 (Lonza) with 1% heat-inactivated, filtered human AB serum (Sigma). Total CD4^+^ T cells were isolated by negative selection using magnetic beads (StemCell Technologies) and incubated with Fixable Viability Dye eFluor 780 (eBioscience) for 15 min at room temperature. After washing in PBS with 0.02% BSA, cells were stained in 5 mL FACS tubes (Falcon) with the fluorochrome-conjugated antibodies used for cell sorting and the BD AbSeq oligo-conjugated antibodies (BD Bioscience), according to the manufacturer’s instructions.

Cell sorting was performed using a BD FACSAria Fusion sorter (BD Biosciences) at 4 °C into 1.5 mL DNA low bind Eppendorf tubes containing 500 μL of X-VIVO with 1% heat-inactivated, filtered human AB serum. Following cell sorting, the three assessed T cell subsets were incubated with Sample Tag antibodies (Sample multiplexing kit; BD Bioscience), washed 3 times in cold BD sample buffer (BD Biosciences) and counted. Samples were then pooled together in equal ratios in 620 μL of cold BD sample buffer at the desired cell concentrations—ranging from 20 to 40 cells/μL for an estimated capture rate of 10,000–20,000 single-cells—and immediately loaded on a BD Rhapsody Cartridge (BD Biosciences) for single-cell capture.

For the in vitro-stimulated condition, sorted CD4^+^ T cell subsets were incubated in 96-well round-bottom tissue culture plates (20,000 cells/well) at 37 °C for 90 min in X-VIVO with 5% heat-inactivated, filtered human AB serum with a PMA and ionomycin cell stimulation cocktail (eBioscience), in the absence of protein transport inhibitors. Cells were harvested into FACS tubes, washed with cold BD sample buffer and further incubated with the BD AbSeq oligo-conjugated antibodies, according to the manufacturer’s instructions. All FACS/sorting and AbSeq antibodies used in this study are listed in Additional file 1: Table S1.

### CD80/86 immunophenotyping

Immunophenotyping of the co-stimulatory molecules CD80/CD86 and CTLA-4 was performed in freshly isolated PBMCs. Cells were initially stained with fluorochrome-conjugated antibodies against surface receptors (see Additional file 1: Table S1) in BD Brilliant Stain Buffer (BD Biosciences) for 30 min at room temperature then washed three times with PBS 0.02% BSA to remove any residual antibody before cell permeabilisation. Fixation and permeabilisation were performed using the FOXP3 Fix/Perm Buffer Set (eBioscience) according to the manufacturer’s instructions. Cells were then immunostained with fluorochrome-conjugated antibodies against intracellular markers (including CTLA-4, where indicated) in BD Brilliant Stain Buffer for 45 min at room temperature.

For the in vitro stimulation assays, total CD4^+^ T cells were isolated by negative selection using magnetic beads (StemCell Technologies) from PBMCs from two of the healthy donors used for ex vivo phenotyping. One million CD4^+^ T cells, resuspended at a concentration of 10^6^ cells/mL, were incubated in X-VIVO with 5% heat-inactivated, filtered human AB serum, 1× GlutaMAX + Pen/Strep in 24-well flat-bottom tissue culture plates (TPP, Techno Plastic Products AG) in the presence of 0, 50 or 500 IU IL-2 (IL-2 stimulation assay), or with αCD3/CD28 beads (1 bead, 3 cells; Dynabeads Human T-activator CD3/CD28, Thermo Fisher) and 0 or 500 IU IL-2 (TCR stimulation assay). Cells were harvested every  four days for immunophenotyping as described for the ex vivo cells; residual cells were restimulated using the same original cell culture conditions. At day 15, a resting condition was also included, whereby CD4^+^ T cells were no longer restimulated with αCD3/CD28 beads.

Immunostained samples were acquired using a BD Fortessa (BD Biosciences) flow cytometer with FACSDiva software (BD Biosciences) and analysed using FlowJo (Tree Star, Inc.). Live, single, CD4^+^ T cells were assessed following exclusion of dead cells based on the Fixable Viability Dye eFluor 780 (eBioscience) and exclusion of CD56^+^, CD14^+^ and CD8^+^ cells.

### Sorting and expansion of regulatory T cells

CD25^hi^CD127^low^ Tregs were flow-sorted from three healthy donors and expanded using the Treg expansion kit (Miltenyi Biotec) in X-VIVO containing 1% heat-inactivated human AB serum, 500 U/mL IL-2 and 50 ng/mL rapamycin (Miltenyi Biotec). Tregs were expanded for cycles of two weeks for up to six additional rounds of re-stimulation in G-rex G10 flasks (Wilson Wolf). Cells were harvested either when they were actively expanding (8 days after re-stimulation) or at the end of the expansion cycle (day 15 after re-stimulation). Treg expansion beads were then removed and cells stored in cell-freezing media for flow cytometry analyses (Sigma) or RLT buffer for mRNA analyses (Qiagen). Regulatory phenotype and proliferative state were confirmed by flow cytometric analysis at the time of sample collection and storage. mRNA expression of CD80 and CD86 was assessed by NanoString (NanoString Technologies), according to the manufacturer’s instructions.

### Tissue dissociation and isolation of CD45^+^ immune cells from duodenal biopsies

For the characterisation of CD45^+^ immune cells from the intestine, duodenal biopsies were cryopreserved in CryoStor CS10 reagent (StemCell) and stored in liquid nitrogen until sample processing. Paired blood-derived PBMCs were processed as described above. The paired duodenal biopsies were thawed at 37 °C in X-VIVO with 1% heat-inactivated, filtered human AB serum then subjected to gentle mechanical dissociation using gentleMACS (Miltenyi Biotec) followed by short 20-min enzymatic dissociation at 37 °C using a very low concentration of Liberase TL (0.042 mg/mL; Sigma), 10 nM HEPES and 1 mg/mL DNase I in X-VIVO with 10% FBS. Following enzymatic dissociation, the biopsies were homogenised using a more vigorous gentleMACS cycle and strained through a 70-μm filter with physical maceration to generate single-cell suspensions. CD45^+^ immune cells were further enriched using a 70/35% Percoll gradient (Sigma). The dissociation protocol and low concentration of Liberase TL enzyme were optimised to show a minimal effect on the degradation of surface protein expression levels, as assessed by flow cytometry. This was critical to ensure maximal sensitivity and specificity of the AbSeq protein quantification in these samples.

Blood- and tissue-derived single-cell suspensions were incubated with Fixable Viability Dye eFluor 780 for 15 min at room temperature, and total CD45^+^ cells were isolated by FACS. Following cell sorting, the individual blood- and tissue-derived subsets were incubated with Fc block reagent (BD Biosciences) and Sample Tag antibodies for 20 min at room temperature. Following three rounds of washing, cells were counted and equal numbers (35,000 cells) of blood- and tissue-derived cells from the same donor were pooled together and incubated with AbSeq antibody mastermix (Additional file 1: Table S1) according to the manufacturer’s instructions. Cells were then washed two times in cold sample buffer, counted and resuspended in 620 μL of cold sample buffer at a final concentration of 40 cells/μL for loading on a BD Rhapsody Cartridge.

### cDNA library preparation and sequencing

Single-cell capture and cDNA library preparation were performed using the BD Rhapsody Express Single-Cell Analysis System (BD Biosciences), according to the manufacturer’s instructions. Briefly, cDNA was amplified—10 cycles for resting cells and 9 cycles for in vitro-stimulated cells—using the Human Immune Response Primer Panel (BD Biosciences), containing 399 primer pairs, targeting 397 different genes. The resulting PCR1 products were purified using AMPure XP magnetic beads (Beckman Coulter), and the respective mRNA and AbSeq/Sample Tag products were separated based on size selection, using different bead ratios (0.7× and 1.2×, respectively). The purified mRNA and Sample Tag PCR1 products were further amplified (10 cycles), and the resulting PCR2 products purified by size selection (1× and 1.2× for the mRNA and Sample Tag libraries, respectively). The concentration, size and integrity of the resulting PCR products were assessed using both Qubit (High-Sensitivity dsDNA Kit; Thermo Fisher) and the Agilent 4200 TapeStation system (High Sensitivity D1000 ScreenTape; Agilent). The final products were normalised to 2.5 ng/μL (mRNA), 0.5 ng/μL (Sample Tag) and 0.275 ng/μL (AbSeq) and underwent a final round of amplification (6 cycles for mRNA and 8 cycles for Sample Tag and AbSeq) using indexes for Illumina sequencing to prepare the final libraries. Final libraries were quantified using Qubit and Agilent TapeStation and pooled (~ 60/38/2% mRNA/AbSeq/Sample Tag ratio) to achieve a final concentration of 5 nM. Final pooled libraries were spiked with 10% PhiX control DNA to increase sequence complexity and sequenced (75 bp paired-end) on HiSeq 4000 sequencer (Illumina).

### Data analysis and QC

The FASTQ files obtained from sequencing were analysed following the BD Biosciences Rhapsody pipeline (BD Biosciences). Initially, read pairs with low quality were removed based on read length, mean base quality score and highest single-nucleotide frequency. The remaining high-quality R1 reads were analysed to identify cell label and unique molecular identifier (UMI) sequences. The remaining high-quality R2 reads were aligned to the reference panel sequences (mRNA and AbSeq) using Bowtie2. Reads with the same cell label, the same UMI sequence and the same gene were collapsed into a single molecule. The obtained counts were adjusted by BD Biosciences-developed error correction algorithms—recursive substitution error correction (RSEC) and distribution-based error correction (DBEC)—to correct sequencing and PCR errors. Cell counts were then estimated, using the second derivative analysis to filter out noise cell labels, based on the assumption that putative cells have much more reads than noise cell labels. Thus, when cells are sorted in the descending order by the number of reads, the inflexion point can be observed on a log-transformed cumulative curve of the number of reads. For the CD45^+^-sorted cells, due to the heterogeneity of the sample, we observed two inflexion points (and two corresponding second derivative minima), and therefore, only cell labels after the second inflexion point were considered noise labels. Barcoded oligo-conjugated antibodies (single-cell multiplexing kit; BD Biosciences) were used to infer the origin of sample (i.e. sorted cell population) and multiplet rate by the BD Rhapsody Analysis pipeline.

The DBEC-adjusted molecule counts obtained from the Rhapsody pipeline were imported, and the expression matrices were further analysed using the R package Seurat 3.0 [11]. Most cells identified as undetermined by the Rhapsody pipeline had a low number of features (mRNA and protein reads). These cells along with other cells with similarly low (< 35) number of features were filtered out. Identified multiplet cells were also filtered out at this stage. A detailed summary of the number of putative captured cells, multiplet rate and number of cells filtered from the analysis in each of the three experiments performed in this study is provided in Additional file 2: Table S2. The resulting matrices were log normalised using the default parameters in Seurat, and the UMI counts were regressed out when scaling data. In this approach, protein (AbSeq) UMI counts were included in the same normalisation along with mRNA UMI counts. To investigate the relative contribution of the protein library to the identified clusters, we also tested two alternative normalisation methods: (i) a hybrid method that integrates protein and mRNA data and uses different normalisation methods for them—for protein data, we use a centred log-ratio (CLR) normalisation, computed independently for each feature, and for mRNA data, we use typical log normalisation—and (ii) using only the mRNA data. Uniform Manifold Approximation and Projection (UMAP) was used for dimensionality reduction. The default number of used dimensions of PCA reduction was increased to 30 based on Seurat elbow plot. For clustering, we increased the default clustering resolution parameter value to 1.2 to obtain a more fine-grained set of clusters. We note that the choice of the clustering parameter can depend on several experimental factors, such as cell number and heterogeneity of the starting cell population. Although we observed relatively stable cluster assignment around the value of the clustering parameter chosen for this study, the optimal parameter may depend on the aim of the analysis and on the desired granularity of the resulting cell clusters. Differential expression analysis was performed using negative binomial generalised linear model implemented in Seurat, and integration of data from multiple experiments was performed using a combination of canonical correlation analysis (CCA) and identification of mutual nearest neighbours (MNN), implemented in Seurat 3.0 [12]. For integrated datasets, the differential expression testing was performed for each integrated dataset, and the *p* values were combined using meta-analysis methods from the Metap R package implemented in Seurat.

The Seurat objects were further converted and imported to the SCANPY toolkit [13] for consecutive analyses. We have computed diffusion pseudotime according to Haghverdi et al. [14] which is implemented within SCANPY and used the partition-based graph abstraction (PAGA) method [15] for formal trajectory inference and to detect differentiation pathways. For visualisation purposes, we discarded low-connectivity edges using the threshold of 0.7. Additionally, we have also performed a pseudotime analysis using another independent method: single-cell trajectories reconstruction (STREAM) [16]. In this case, to generate appropriate input files, the Seurat objects were subsampled to include *N* = 2500 cells. The values of other parameters not mentioned here were set to default.

### Detection of FOXP3 expression in Tregs using whole-transcriptome scRNA-seq data

Expression of *FOXP3* was assessed in two publicly available 10× Genomics datasets combining 3′ mRNA and surface protein expression: a 10k PBMC dataset generated using the v3 chemistry (7865 cells passing QC, with an average of 35,433 reads per cell for the mRNA library) and a 5k PBMC dataset using the NextGEM chemistry (5527 cells passing QC, with an average of 30,853 reads per cell for the mRNA library; available at https://support.10xgenomics.com/single-cell-gene-expression/datasets/). Treg and non-Treg gates were delineated using the filtered cell matrixes with SeqGeq™ (FlowJo, Tree Star, Inc.), using the same strategy employed to sort the CD127^low^CD25^hi^ Treg population in this study. FOXP3^+^ cells were defined as cells expressing one or more copy (UMI) of *FOXP3*.

## Results

### Simultaneous protein quantification increases the power of scRNA-seq to dissect the functional heterogeneity of human CD4^+^ T cells

**Fig. 1:**
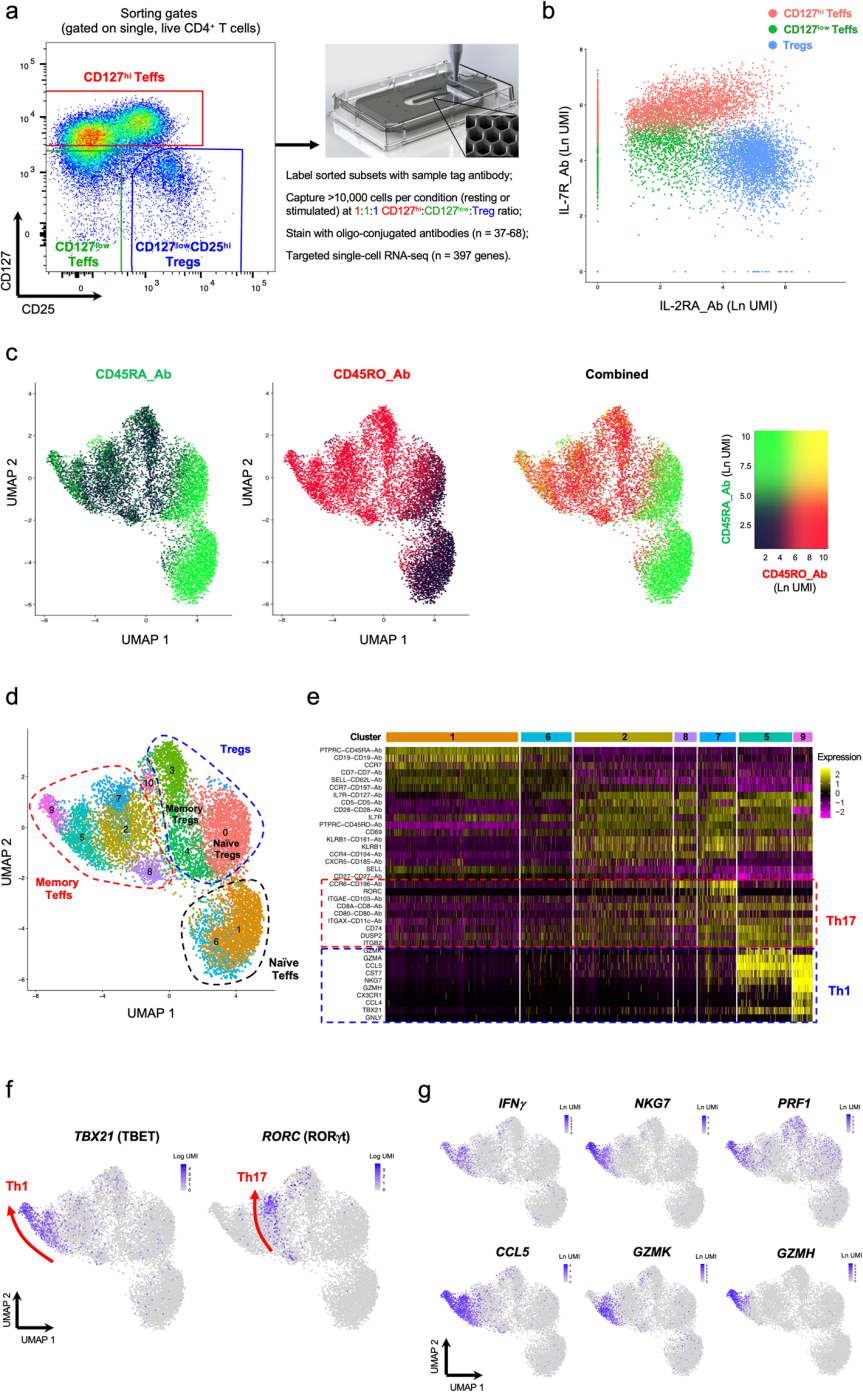
Combined single-cell transcriptional and proteomics immunophenotyping provides a high-resolution map of human primary CD4^+^ T cells in the blood. **a** Summary of the experimental workflow. FACS plot depicting the sorting strategy for the isolation of the three assessed CD4^+^ T cell populations. **b** Two-dimensional plot depicting the expression of IL-7R and IL-2RA at the protein level using oligo-conjugated antibodies (AbSeq). Cells are coloured according to their respective sorting gate, as assessed using oligo-conjugated sample-tagging antibodies. **c** Uniform Manifold Approximation Projection (UMAP) plot depicting the clustering of all captured CD4^+^ single cells using the combined proteomics and transcriptomics data. Expression levels of the CD45RA (black to green) and CD45RO (black to red) isoforms obtained using the AbSeq technology are depicted in the plot. **d** UMAP plot depicting the clustering of resting primary CD4^+^ T cells (*n* = 9708) isolated from the blood of a systemic lupus erythematosus (SLE) patient. Dashed lines delineate the naive Teff (black), memory Teff (red), and Treg (blue) clusters, annotated manually based on their respective protein and mRNA expression profiles. **e** Heatmap displaying the top 10 differentially expressed genes in each resting CD4^+^ Teff cluster. **f** UMAP plots depicting the expression of the CD4^+^ T cell lineage-defining transcription factors TBET (Th1) and RORγt (Th17) in resting CD4^+^ T cells. Arrows recapitulate the identified axis of Th1 and Th17 differentiation and are supported both on the gradient of expression of the respective lineage-restricted transcription factors (TBET and RORγt, respectively) and on the developmental trajectories identified by the pseudotime analysis depicted in Fig. 3. **g** Expression of the effector-type cytokine transcripts *IFNG*, *NKG7*, *PRF1*, *CCL5*, *GZMH* and *GZMK* in resting CD4^+^ T cells

In this study, we wanted to investigate the power of a unified high-throughput experimental workflow combining targeted scRNA-seq and the quantification of protein expression at the single-cell level, to dissect the heterogeneity of human primary CD4^+^ T cells in the blood. To address this question, we initially profiled the expression of 397 genes at the mRNA level, coupled with 37 protein targets (Additional file 1: Table S1) using the BD AbSeq technology, in CD4^+^ T cells isolated from the blood of an SLE patient. To enrich for the relative distribution of two less abundant CD4^+^ T cell subsets: (i) CD127^low^CD25^hi^ T cells, predominantly containing the Treg population; and (ii) CD127^low^CD25^low^ T cells, containing a subset of non-conventional CD25^low^FOXP3^+^ Tregs previously characterised in autoimmune patients [17], we devised a FACS-sorting strategy to isolate and profile equal numbers of cells from the three defined T cell subsets (Fig. 1a). Following sorting, cells from each subset were labelled with a barcoded oligo-conjugated antibody (Sample Tag) prior to cell capture—a method related to the recently described cell-hashing technique [18]—to identify their original sorting gate and to assess the frequency of cell multiplets obtained in this experiment.

A total of 9898 captured cells passed the initial quality control (QC), of which a small proportion (1.9%; Additional file 2: Table S2) were assigned as multiplets and excluded from the analysis. Of note, we observed complete sequencing saturation of the mRNA library, assessed as the number of cDNA molecules with a novel unique molecular identifier (UMI) identified with increasing sequencing coverage, for a read depth of > 2700 reads/cell (Additional file 3: Figure S1a). In contrast, we obtained approximately 80% sequencing saturation at a read depth of > 6000 reads/cell for the AbSeq library (Additional file 3: Figure S1b). This is illustrated by the large dynamic range of expression of the protein targets, reaching up to thousands of unique copies in cells displaying higher levels of expression (Additional file 3: Figure S1c). Of note, the median expression of most assessed proteins, including all those that are known not to be expressed on CD4^+^ T cells, was zero copies (Additional file 3: Figure S1c), which demonstrates the high specificity of the AbSeq system. To test the sensitivity of this targeted approach to detect lowly expressed genes at the mRNA level at these sequencing coverages compared to whole-transcriptome scRNA-seq methods, we quantified the frequency of cells expressing at least one copy of *FOXP3* within the Treg population. To standardise this comparison between studies, we took advantage of two publicly available PBMC datasets from 10× Genomics, with deep paired cell-surface protein expression data, and delineated the CD127^low^CD25^hi^ Treg population using the same sorting strategy employed here (Additional file 3: Figure S2a,b). While *FOXP3* was detected in only 23.2% and 18.3% of Tregs in whole-transcriptome datasets (using the v3 and NextGEM chemistries, respectively), it was detected in 68.3% of Tregs using the targeted approach described in this manuscript (Additional file 3: Figure S2c). A similar difference was observed within the non-Treg gate, although as expected, the frequency of FOXP3^+^ cells was substantially lower (Additional file 3: Figure S2c). In addition, we observed different *FOXP3* UMI count distributions, with a distinct skew towards higher UMI counts using the targeted approach (Additional file 3: Figure S2d). These data demonstrate the sensitivity of targeted scRNA-seq to detect lowly expressed genes, even at a fraction of the sequencing coverage (approx. 1/10th), therefore allowing to allocate additional sequencing resources to the protein expression libraries, which display much higher dynamic range of expression.

To further test the sensitivity of the AbSeq protein measurements, we next generated a two-dimensional plot depicting the AbSeq expression of IL-2RA (CD25) and IL-7R (CD127), which recapitulated the flow cytometric profile obtained with the same two markers used for the sorting of the assessed T cell subsets (Fig. 1b). Furthermore, by overlaying the Sample Tag information, we were able to confirm that the expression profiles of CD127 and CD25 mimicked the sorting strategy precisely for all three sorted CD4^+^ T cell subsets (Fig. 1b), therefore illustrating the highly quantitative nature of the protein measurements. We note that in our study, the read depth devoted to the protein library (approx. 6000 reads/cell) was insufficient to reveal the complete spectrum of CD127 and CD25 expression, resulting in reduced power to fully resolve CD25^low/int^ T cells, when compared to flow cytometry. Although increased sequencing coverages in our experiment would provide only minimal gain for the functional characterisation of single cells and clustering, they are necessary to describe the complete dynamic range of protein expression, especially for highly expressed proteins such as CD127 and CD25, thereby leading to similar sensitivity between molecular flow cytometry, as previously demonstrated for CITE-seq [9], REAP-seq [10] and BD AbSeq [19].

Next, we performed unsupervised Louvain clustering combining the mRNA and protein expression data and visualised the clusters in a two-dimensional space using Uniform Manifold Approximation and Projection (UMAP) [20]. One of the main discriminators of functional differentiation in CD4^+^ T cells is the acquisition of a memory phenotype in response to antigen stimulation, typically marked by the expression of CD45RA on naive cells and CD45RO on memory cells. However, because these are splice isoforms of the same gene (*PTPRC*; CD45), discrimination cannot be achieved using UMI-based scRNA-seq systems targeting the 3′ or 5′ ends of the transcript. By measuring the expression of the two isoforms at the protein level, we were able to identify a marked expression gradient associated with a gradual loss of CD45RA and concomitant gain of CD45RO along the first component of the UMAP plot, indicating that the acquisition of a memory phenotype is indeed the main source of biological variation driving the clustering of CD4^+^ T cells in both the Teff and Treg compartments (Fig. 1c). One notable exception was the re-expression of CD45RA in the most differentiated memory cells (Fig. 1c). This observation is consistent with the phenotype of differentiated effector memory CD4^+^ T cells that re-express CD45RA (TEMRAs) [21] and illustrates the power of this highly multiparametric approach to identify subtle alterations in CD4^+^ T cell states, while mitigating the potential issue of cell-type misclassification based on a few prototypical markers such as CD45RA/RO.

In this study, we have opted to integrate the mRNA and protein data using standard normalisation methods, which have been developed for traditional scRNA-seq technologies. In the first approach, we do not differentiate between mRNA and protein libraries. To further investigate the relative contribution of the protein library to the underlying clustering, we also applied two alternative normalisation methods: (i) a hybrid method involving independent normalisation of protein and mRNA libraries and (ii) using only the mRNA library for clustering (Additional file 3: Figure S3a,b). Overall, we observed good concordance between the resulting cell clusters and their assigned functional annotation regardless of the normalisation method used, although small differences could be observed in the resolution of the smaller clusters (Additional file 3: Figure S3c,d). These data suggest that for this dataset, containing a relatively small number of protein markers, this issue does not significantly alter the functional annotation of the clusters and the interpretation of the results. Nevertheless, the transcriptomics-only approach provided slightly inferior cell-cluster resolution and spatial separation of the naive-memory differentiation trajectory, indicating the added value of protein data for the functional annotation of the clusters. With the growing popularity of molecular cytometry and increasing number of proteins assessed, it will be important to consider the effects of data normalisation to the interpretation of the results, and therefore, further work is warranted to develop better methods to integrate multi-omics datasets.

### Single-cell mRNA and protein immunophenotyping identifies distinct trajectories of CD4^+^ T cell differentiation in blood

Integration of the multiparametric transcriptional and proteomics data identified discrete clusters of CD4^+^ T cells along the naive/memory differentiation axis (Fig. 1d). We observed an increased number of clusters within the memory compartment, marked by the differential expression of defined sets of signature genes (Fig. 1e and Additional file 3: Figure S4a), which was consistent with the increased functional heterogeneity in differentiated CD4^+^ T cell subsets. Moreover, we observed that the expression of the canonical Th1 (*TBX21*, encoding TBET) and Th17 (*RORC*, encoding RORγt) lineage-defining transcription factors was restricted to specific clusters within the memory Teff (mTeff) population (Fig. 1f), indicating that these clusters are highly enriched for Th1 and Th17 Teffs. More importantly, we observed a distinct gradient of expression of these transcription factors. Consistent with this gradient of functional differentiation, we observed marked co-expression of canonical Th1 effector-type molecules and TBET (Fig. 1f), revealing a subset of highly activated Th1 T cells with a putative cytotoxic profile in the blood of this SLE patient. Similarly, a gradient of expression of Th17 signature genes, including *RORC*, could be observed from clusters 8 to 7 (Fig. 1e, f), indicating a trajectory of Th17 differentiation.

In addition to resting CD4^+^ T cells, we also profiled the same subsets of cells following short in vitro stimulation (90 min) with PMA + ionomycin, to assess cell type-specific cytokine production. Similarly to the resting condition, in vitro-stimulated CD4^+^ T cells formed discrete clusters along the naive-memory differentiation axis (Additional file 3: Figure S5a,b). Furthermore, we observed a consistent induction of expression of Th1 (IFNγ) and Th17 (IL-22) type cytokines that were restricted to the respective Th1 and Th17 clusters (Additional file 3: Figure S5c,d). Although primers for the Th2 transcription factor gene *GATA3* were not included in this assay, therefore precluding the annotation of Th2 cells in resting CD4^+^ T cells, we noted that in vitro stimulation revealed a distinct cluster of Th2 mTeffs cells marked by the expression of Th2-type cytokines, such as IL-13 (Additional file 3: Figure S5e), IL-4, IL-5 and IL-9 (Additional file 3: Figure S4b).

**Fig. 2:**
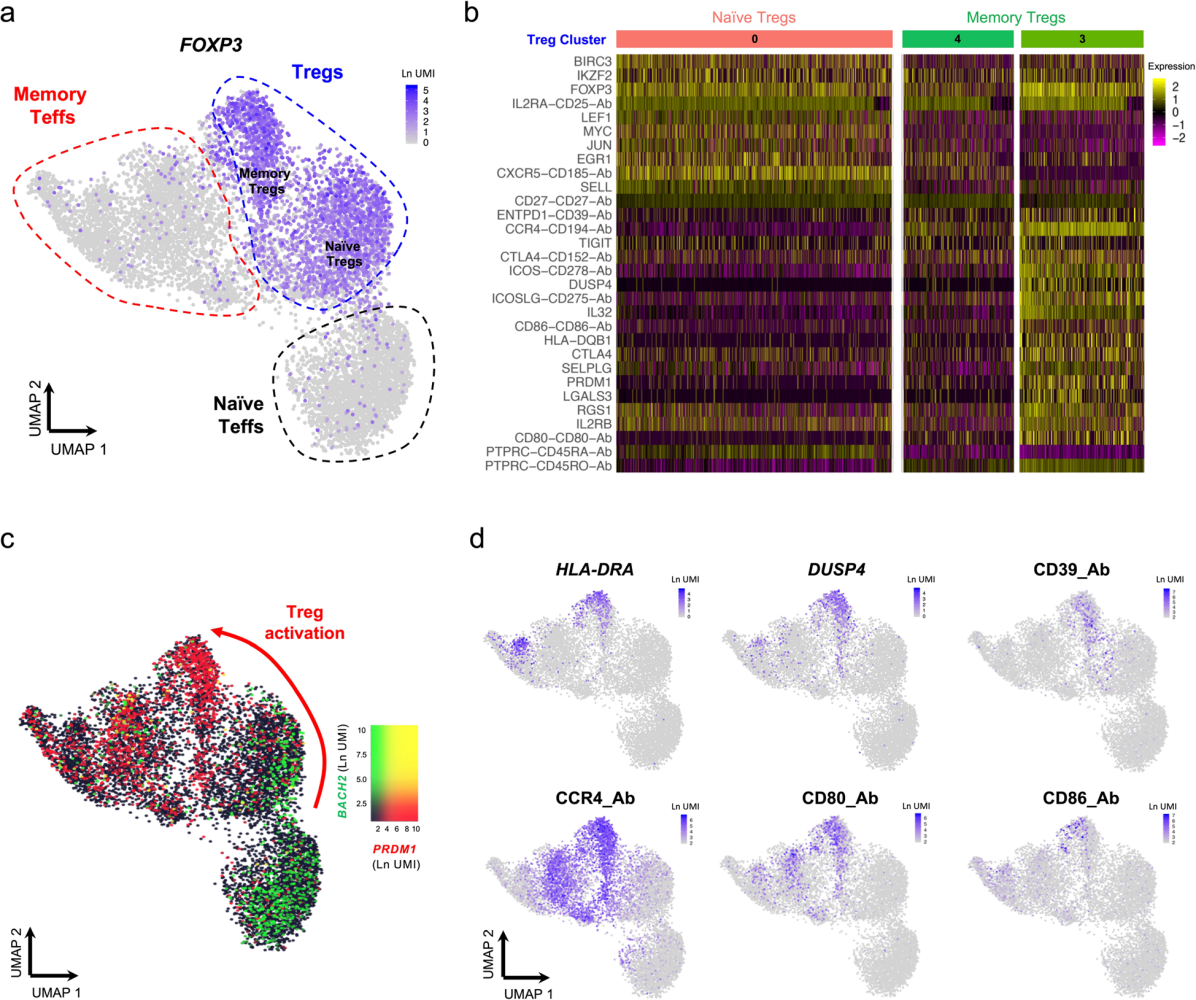
Integrated single-cell targeted multi-omics approach identifies a trajectory of human CD4^+^ regulatory T cell (Treg) activation. **a** UMAP plot depicting the expression of the canonical Treg transcription factor FOXP3 in the identified resting CD4^+^ T cell clusters. Naive and memory Treg clusters are annotated as shown in Fig. 1c. **b** Heatmap displaying the top 10 differentially expressed genes within the three identified resting Treg clusters, depicted in Fig. 1d. **c** UMAP plot depicting the overlaid expression of the CD4^+^ T cell transcription factors *BACH2* (black to green) and *PRDM1* (encoding BLIMP-1; black to red). **d** Illustrative examples of the expression of highly differentially expressed genes within the cluster of activated Tregs (cluster 3), including *HLA-DRA* and *DUSP4* at the mRNA level and CD39, CCR4, CD80 and CD86 at the protein level

Recently, several scRNA-seq studies have refined our understanding of the heterogeneity of CD4^+^ Tregs and their functional adaptation in tissues, in both mice and humans [22, 23]. Given the sorting strategy used in this study, we were able to significantly enrich our CD4^+^ T cell dataset for Tregs, which are highly enriched within the CD127^low^CD25^hi^ population. Consistent with this enrichment strategy, we identified a large Treg population, marked by the expression of the transcription factor FOXP3 and other classical Treg signature genes, including HELIOS (encoded by *IKZF2*), IL-2RA, CTLA-4 or TIGIT (Fig. 2a, b). In agreement with their Treg-specific transcriptional programme, we found a marked suppression of IL-2 transcription in Tregs following in vitro stimulation (Additional file 3: Figure S5f). We also identified a naive Treg cluster (cluster 0, Fig. 1d) marked by elevated expression of the canonical naive T cell marker CD45RA and concurrent low expression of CD45RO (Fig. 1c). Furthermore, naive Tregs also displayed elevated expression of additional naive T cell signature genes, including CD62L (*SELL*), CCR7 and CD7 (Additional file 3: Figure S4a), highlighting a population of thymically derived naive Tregs that have been previously characterised in the thymus and cord blood, decreasing with age in adults [24]. In particular, we found that the differentiation of Tregs from a naive to memory phenotype was strongly associated with the expression of two transcription factors: BACH2 and BLIMP1 (encoded by *PRDM1*). These two key transcription factors displayed a distinct mutually exclusive expression pattern, with high expression of *BACH2* mRNA in naive cells, declining gradually—with a concomitant gradual increase in *PRDM1* expression—along the naive-memory differentiation axis (Fig. 2c). The gradual increase of *PRDM1* expression was found to be strongly associated with the expression of Treg activation markers such as *HLA-DRA*, *DUSP4* and *CD39* (Fig. 2d), and revealed a trajectory of Treg activation in resting primary CD4^+^ T cells. These data suggest that the transcriptional interplay between BACH2 and BLIMP-1 is critical to regulate the differentiation of memory Treg subsets, which is in agreement with previous data in both mice and humans [22]. The dynamic interplay of *BACH2* and *PRDM1* in the differentiation of Tregs was even more pronounced following in vitro stimulation (Additional file 3: Figure S6), which further supports the hypothesis that they are primary regulators of the transcriptional programme associated with the differentiation of activated Tregs in humans, in response to antigen stimulation.

**Fig. 3:**
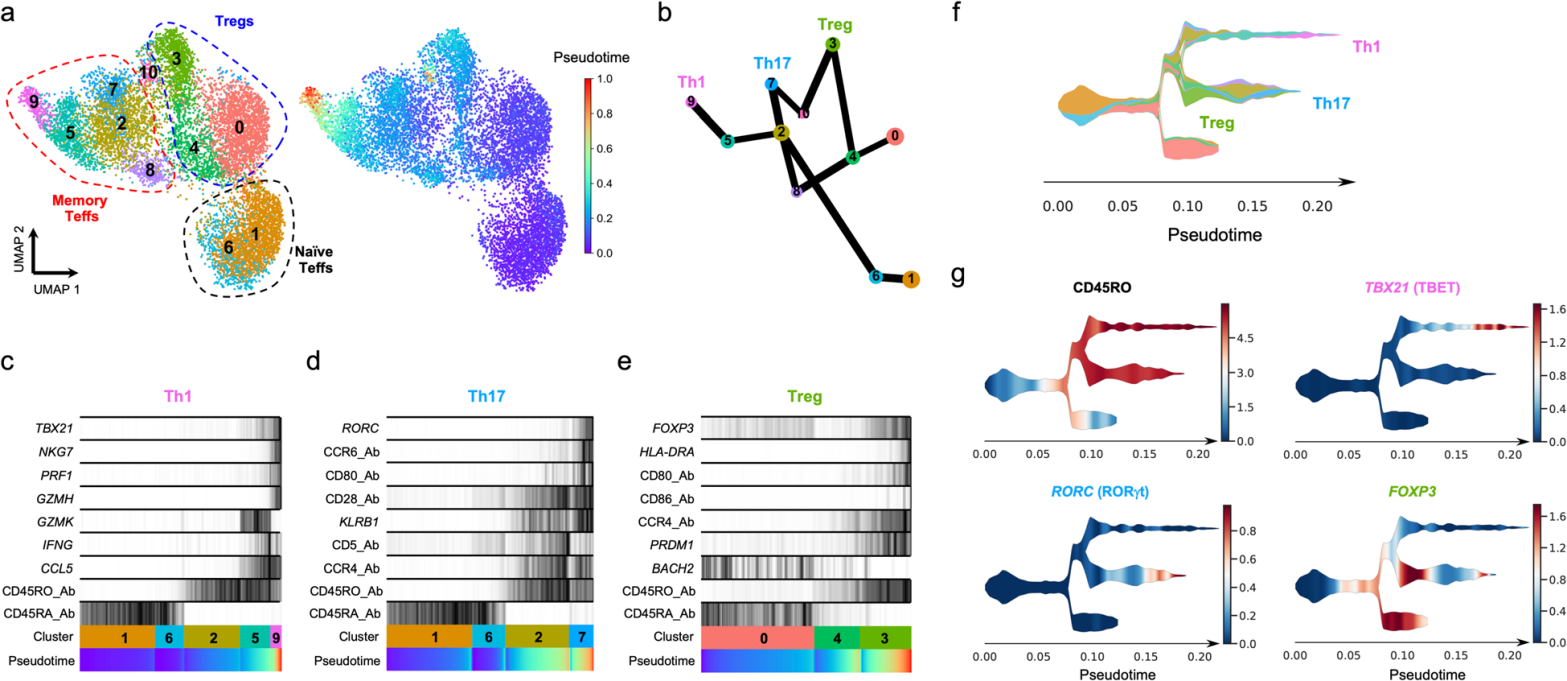
Pseudotime analysis reveals distinct trajectories of CD4^+^ T cell differentiation in vivo. **a** UMAP plots depicting the inferred diffusion pseudotime of each single cell in the identified T cell clusters. **b** Graph reconstructing the developmental trajectories between the identified T cell clusters. Edge weights represent confidence in the presence of connections between clusters. The analysis was performed in the combined transcriptional and proteomics data using the partition-based graph abstraction (PAGA) method. **c**–**e** Reconstructed PAGA paths for the differentiation of the identified Th1 (**c**), Th17 (**d**) and Treg (**e**) lineages. Expression of the lineage-specific transcription factors and selected differentially expressed genes is depicted for each trajectory. **f** Schematic representation of the identified lineage differentiation trajectories using the single-cell trajectories reconstruction (STREAM) method. Colour code corresponds to the cluster assignment depicted in **a**. **g** Expression of the memory-associated CD45RO isoform and the lineage-specific transcription factors *TBX21* (encoding TBET), *RORC* (encoding RORγt) and *FOXP3* is depicted along the identified developmental branches

Statistical methods are currently being developed to identify and reconstruct developmental trajectories from heterogeneous scRNA-seq datasets using pseudotime analysis. To validate our findings, we next applied the recently developed partition-based graph abstraction (PAGA) method [15] to reconstruct the developmental trajectories in our dataset. Consistent with our previous findings, the pseudotime analysis revealed a gradient of T cell differentiation along the naive-memory differentiation axis, which lead to the identification of three distinct differentiation pathways associated with the acquisition of a Th1, Th17 or Treg phenotype (Fig. 3a, b). These identified differentiation trajectories were associated with gradually increased expression of the lineage-specific transcription factors TBET, RORγt and FOXP3, respectively, which regulate the transcriptional programme associated with the respective T cell lineages (Fig. 3c–e). In particular, we confirmed a very distinct and gradual differentiation of the Th1 lineage in this SLE patient, leading to the temporal acquisition of activated Th1 cells expressing IFN-γ in cluster 5 and the terminal differentiation of a subset with a cytotoxic profile (cluster 9). Of note, this analysis identified cluster 2 as an intermediate memory Teff cell state, leading to the differentiation of either Th1 (cluster 5 and 9) or Th17 (cluster 7) T cells. Moreover, the pseudotime analysis also recapitulated the Treg differentiation trajectory from naive Tregs (cluster 0) to activated memory Tregs (cluster 3), which was regulated by the mutually exclusive expression of the BACH2 and BLIMP-1 transcription factors (Fig. 3e). An intriguing observation was the identification of cluster 8 representing a potential intermediate T cell state on a Treg-Th17 developmental pathway, which is consistent with the plasticity and putative common co-evolutionary origin between these two lineages [25].

The identification of the temporal differentiation of these T cell lineages was also recapitulated using single-cell trajectories reconstruction, exploration and mapping (STREAM) [16], another method that has been developed to visualise developmental trajectories using multi-omics data (Fig. 3f). Further supporting a putative common developmental pathway of Treg and Th17 cells, STREAM analysis identified FOXP3^+^ memory Tregs (mTregs) as a less differentiated T cell state, which shares a developmental trajectory with differentiated RORγt^+^ Th17 cells, with cluster 8 representing an intermediate transitional cell state in this trajectory (Fig. 3g). These data illustrate not only the potential of the targeted scRNA-seq approach to sensitively quantify lowly expressed transcription factor genes, but also highlight the power of this integrated multi-omics approach to identify subtle cell-state transitions and underlying differentiation trajectories in resting human primary T cells.

### Protein expression of CD80 and CD86 marks a subset of recently activated CD4^+^ Tregs in circulation

**Fig. 4:**
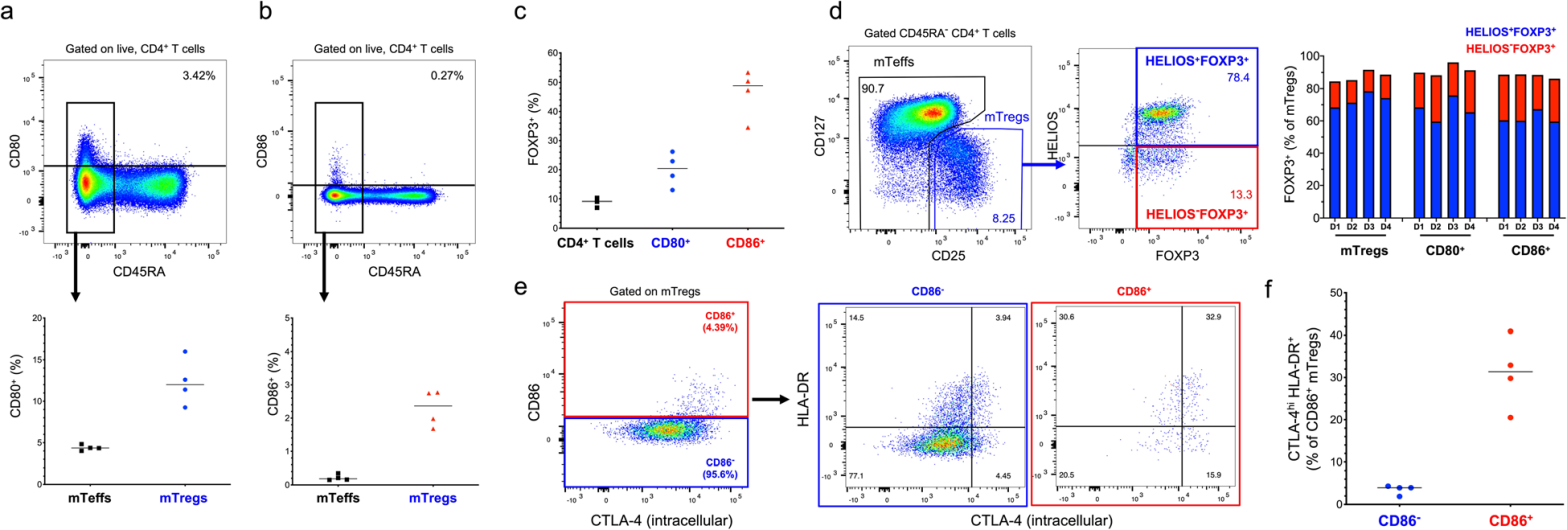
Surface expression of the co-stimulatory molecules CD80 and CD86 marks a subset of activated Tregs in vivo. **a**, **b** Expression of the B7 molecules CD80 (**a**) and CD86 (**b**) was assessed ex vivo by flow cytometry in CD4^+^ T cells from four healthy donors. Scatter plots depict the frequency (median) of CD80^+^ and CD86^+^ T cells within the CD45RA^−^ Teff (mTeffs; black) and CD45RA^−^ CD127^low^CD25^+^ Treg (mTregs; blue) populations. **c** Scatter plot depicts the frequency (median) of FOXP3^+^ cells within total (black), CD80^+^ and CD86^+^ CD4^+^ T cells. **d** Gating strategy for the delineation of the Treg subsets according to the intracellular expression of the canonical Treg transcription factors FOXP3 and HELIOS. Scatter plot depicts the frequency of HELIOS^+^FOXP3^+^ (blue) and HELIOS^−^FOXP3^+^ (red) subsets in each of the four donors within total mTregs, CD80^+^ mTregs and CD86^+^ mTregs. **e** Co-expression of CD86 and the CD4^+^ T cell activation markers CTLA-4 and HLA-DR was assessed by flow cytometry in CD86^+^ (red) and CD86^−^ (blue) mTregs. **f** Scatter plots depict the frequency (median) of CTLA-4^hi^ HLA-DR^+^ cells within CD86^+^ and CD86^−^ mTregs. Expression of CTLA-4 was assessed by intracellular immunostaining

A feature of the most activated mTreg cluster (cluster 3) was the marked increased expression of the B7 proteins CD80 (B7.1) and CD86 (B7.2; Fig. 2b, d), two T cell co-stimulatory molecules usually expressed in antigen-presenting cells (APCs). These findings were recapitulated on the pseudotime analysis, which identified CD80/CD86 protein expression as markers of the temporal Treg differentiation trajectory (Fig. 3e). Although we observed virtually no detectable expression of either *CD80* or *CD86* at the mRNA level in either resting or in vitro-stimulated CD4^+^ T cells (Additional file 3: Figure S7a,b), previous reports have demonstrated endogenous expression of both genes in human CD4^+^ T cells upon activation [26–28]. Furthermore, an increasing body of work points to a functional role of both B7 proteins in T cell function [29]. Consistent with our AbSeq data, we detected the expression of CD80 and CD86 by flow cytometry on the surface of a small proportion resting CD4^+^ T cells isolated from the blood of four healthy donors (Fig. 4a, b). The expression of the B7 proteins was restricted to the CD45RA^−^ memory compartment and showed predominant expression in Tregs (Fig. 4a, b). In agreement with these data, a significant proportion of CD80^+^, and especially CD86^+^ T cells, displayed co-expression of the Treg transcription factor FOXP3 (Fig. 4c). Furthermore, both CD80^+^ and CD86^+^ mTregs displayed normal profiles of FOXP3 and HELIOS expression, supporting a bona fide Treg phenotype; although we noted an increased frequency of the HELIOS^−^FOXP3^+^ subset within B7^+^ Tregs (Fig. 4d). Notably, we detected co-expression between CD86 and the activation markers CTLA-4 and HLA-DR, as indicated by the increased frequency of CTLA-4^hi^ HLA-DR^+^ cells within CD86^+^ mTregs (Fig. 4e, f). In contrast to CD86, the co-expression between CD80 and CTLA-4 on mTregs was less pronounced, although still displaying preferential co-expression with the activation markers CTLA-4 and HLA-DR in mTregs (Additional file 3: Figure S7c,d). Taken together, these data support our AbSeq data and identify CD80 and especially CD86 as a specific marker of activated Tregs in circulation.

### In vitro activation drives endogenous expression of CD80 and CD86 expression in human CD4^+^ T cells

**Fig. 5:**
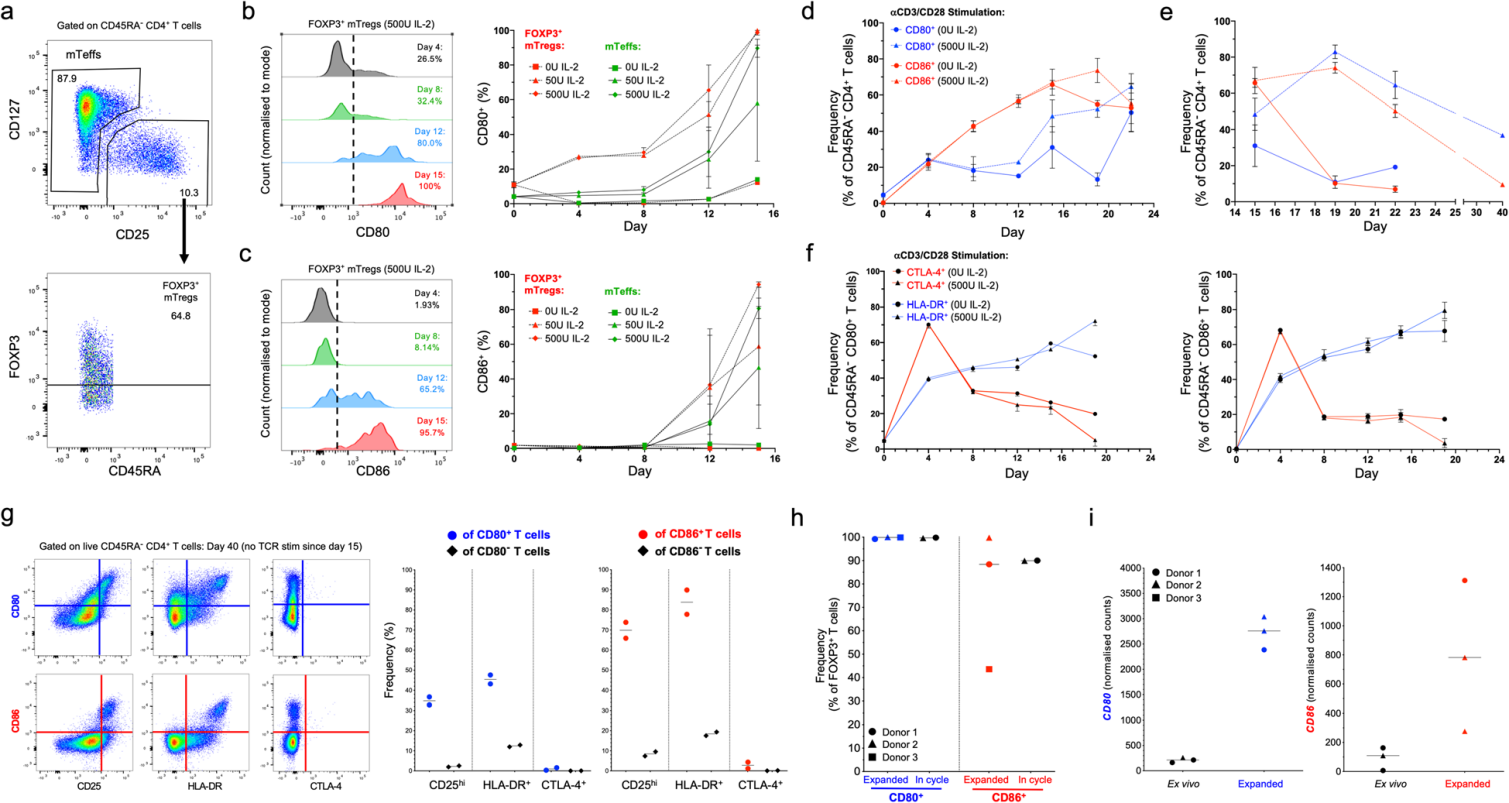
Acquisition and maintenance of B7 molecule expression in CD4^+^ T cells in vitro is dependent on IL-2 signalling. **a** Gating strategy for the delineation of the mTeff and FOXP3^+^ mTreg populations. **b**, **c** Expression of CD80 (**b**) and CD86 (**c**) was assessed by flow cytometry in purified CD4^+^ T cells from two healthy donors incubated in vitro for up to 15 days in the presence of 0 U (square), 50 U (triangle) or 500 U (diamond) of IL-2. Single-parameter histograms represent the expression of CD80 and CD86 in mTreg incubated with 500 U IL-2 from a representative donor. Plots summarise the expression (median and 95% CI of the median) of the markers during the course of the experiment in FOXP3^+^ mTregs (red) and mTeffs (green). **d** Data shown depicts the frequency (median and 95% CI of the median) of CD80^+^ (blue) and CD86^+^ (red) cells within CD45RA^−^ CD4^+^ mTeffs. Data was obtained following in vitro stimulation with αCD3/CD28 beads (one bead to three cells ratio) every four days for two weeks in the presence (500 U; solid line) or absence (dashed line) of IL-2. **e** Frequency of CD80^+^ (blue) and CD86^+^ (red) cells within mTeffs was also assessed up to day 40 in resting cells, which were no longer re-stimulated with αCD3/CD28 after day 15. **f** Plots depict the frequency of membrane-bound CTLA-4^+^ (red) and HLA-DR^+^ (blue) within CD80^+^ and CD86^+^ mTeffs during the course of the experiment. **g** Immunophenotypic characterisation of the activation markers CD25, HLA-DR and CTLA-4 on B7^+^ mTeffs at day 40. Plots depict the frequency (median) of cells expressing these markers within CD80^+^ and CD80^−^ mTeffs (blue; left panel) and CD86^+^ and CD86^−^ (red; right panel) mTeffs, respectively. **h** Frequency (median) of CD80^+^ (blue) and CD86^+^ (red) cells in flow-sorted CD127^low^CD25^hi^ Tregs from three healthy donors, activated in vitro under specific Treg expansion conditions. B7 molecule expression was assessed by flow cytometry in the FOXP3^+^ T cells. For two of the donors, B7 molecule expression was also assessed for cells in the cycle, harvested in the middle of an expansion cycle (eight days after αCD3/CD28 restimulation). Tregs were expanded for between three (D3; square) and six (D1 and D2; circle and triangle, respectively) rounds of re-stimulation. **i** Expression of CD80 and CD86 was also determined at the mRNA level (NanoString) on the sorted Tregs ex vivo or following in vitro expansion

Owing to the high sensitivity of Tregs to IL-2, we next investigated the dynamics of endogenous B7 protein acquisition and its dependence on IL-2 signalling in purified CD4^+^ T cells from two healthy donors incubated in vitro for up to two weeks in the presence or absence of IL-2. Incubation with IL-2 was found to be sufficient to induce the upregulation of CD80 and CD86 expression in the absence of TCR stimulation and was mostly pronounced in FOXP3^+^ mTregs, which express higher levels of IL-2RA than mTeffs (Fig. 5a–c). Of note, the expression of CD80 was upregulated earlier and increased until day 15, where the majority of CD4 T cells were CD80^+^, while CD86 expression was only robustly detected from day 12 (Fig. 5b, c). In addition, we observed some level of inter-individual variation associated with the initial timing of CD86 acquisition, which was more rapid in one of the two assessed donors. In the absence of IL-2, the majority of cells died before day 15.

In contrast to incubation with IL-2 alone, CD4^+^ T cells activated in vitro with TCR stimulation (using αCD3/CD28-conjugated beads) showed robust proliferative capacity, as assessed by increased cell numbers, and increased expression of both B7 molecules in the CD45RA^−^ memory compartment, which reached maximal expression between days 15 and 20 post-stimulation (Fig. 5d and Additional file 3: Figure S7e). No systematic differences were observed in T cells cultured in the presence or absence of IL-2, which was likely due to the high levels of IL-2 production by activated Teffs in this model. However, if cells were not TCR re-stimulated at day 15, we observed a rapid downregulation of CD80 and CD86 expression in cells incubated in the absence of IL-2. In sharp contrast, expression of both B7 proteins in mTeffs was stably maintained for up to 7 days when cells were incubated with high doses (500 U) of IL-2 (Fig. 5e). The acquisition of B7 molecules was associated with T cell activation and was particularly pronounced for CD86 in cells expressing the T cell activation markers CTLA-4 and HLA-DR (Additional file 3: Figure S7f,g). Of note, we observed a distinct pattern of co-expression of these two activation markers in CD80^+^ and CD86^+^ mTeffs. CTLA-4 expression on the cell surface was rapidly and transiently expressed on B7^+^ mTeffs, peaking at day 4, while HLA-DR expression gradually increased over time, showing striking co-expression with both CD80 and CD86 at the later stages of activation (Fig. 5f). In the presence of IL-2, co-expression with CD25 and HLA-DR could be observed in resting cells as late as day 40 post-stimulation, particularly in CD86^+^ T cells. In contrast, membrane-bound CTLA-4 expression was virtually absent at this time point, although the few CTLA-4^+^ cells remaining co-expressed both B7 molecules (Fig. 5g). These data not only demonstrate the stability of B7 protein expression on CD4^+^ T cells following activation, but could also provide a rationale for the observed restricted expression of CD80, and especially CD86, in primary T cells by AbSeq.

The increased proliferative capacity of mTeffs in this model precluded the investigation of the effects of TCR-induced in vitro activation on B7 upregulation in Tregs. To address this question, we next investigated the expression of B7 molecules in flow-sorted CD127^low^CD25^hi^ Tregs activated in vitro under conditions that promote Treg expansion. Similarly to total CD4^+^ T cells, we observed that expanded Tregs showed very high expression of both B7 molecules, which was maintained in cycling cells (assessed as Ki-67^+^), harvested 8 days after αCD3/CD28 restimulation (Fig. 5h). The expression of B7 molecules in expanded Tregs was also confirmed at the mRNA level (Fig. 5i). Given the absence of APCs in these in vitro activation models, these data strongly support an endogenous upregulation of B7 molecules by CD4^+^ T cells in response to activation.

### Multi-omics immunophenotyping identifies a rare subset of circulating CCR9^+^ T cells expressing immune checkpoint molecules

**Fig. 6:**
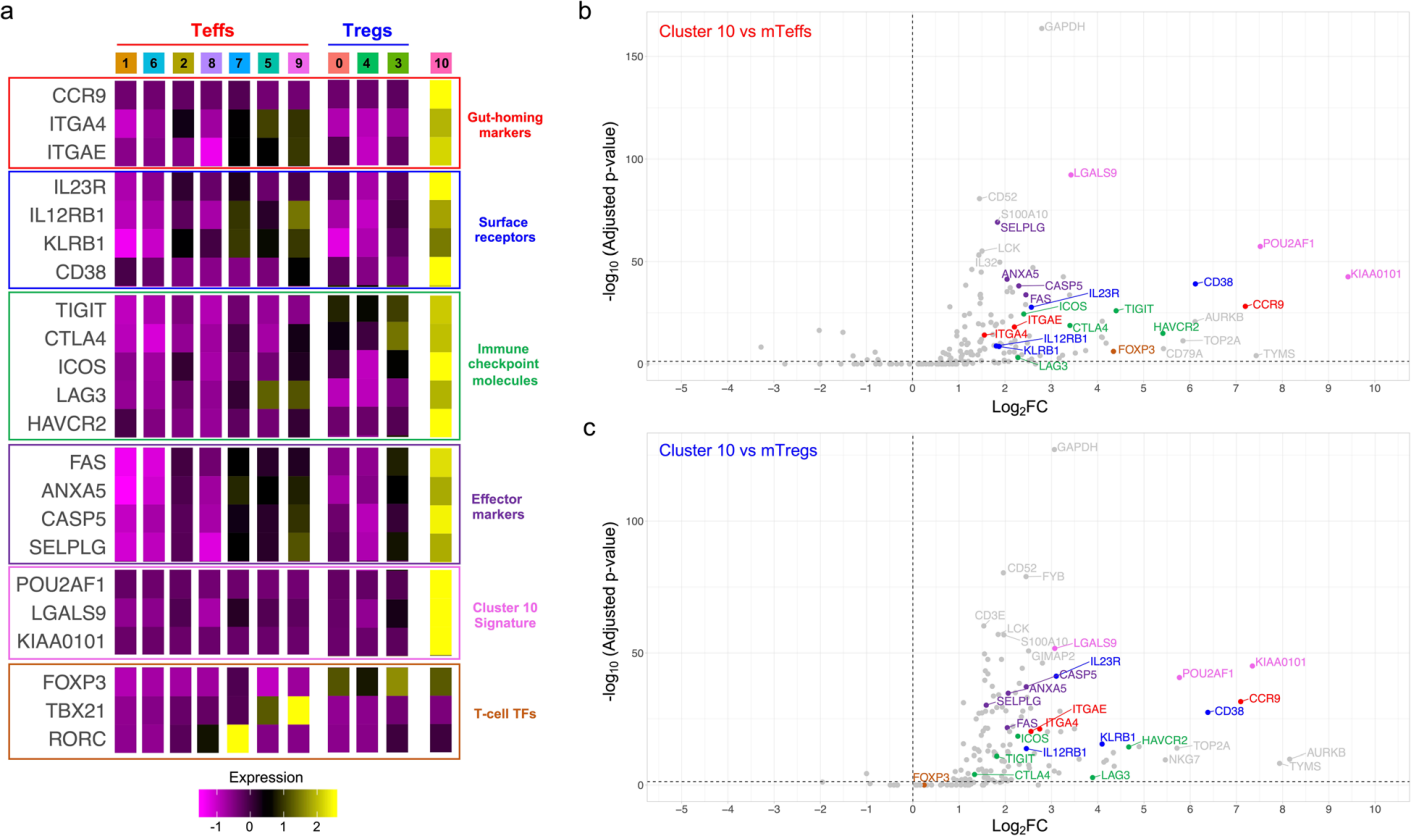
Characterisation of a rare subset of circulating CCR9^+^ T cells with putative immunomodulatory properties. **a** Heatmap depicts the average scaled expression in each identified T cell cluster of selected differentially expressed genes in cluster 10. Markers are grouped according to their functional annotation into gut-homing markers (red), surface receptor (blue), immune checkpoint molecules (green), T cell effector markers (purple), cluster 10 signature genes (pink) and T cell transcription factors (TFs; orange). **b**, **c** Volcano plots depict the differential expression of the assessed mRNA transcripts between cluster 10 and either the mTeff (**b**) or mTreg (**c**) clusters. Colour coding depicts the functional annotation assigned in **a**

Another example of a rare T cell population that we were able to identify in circulation using this multimodal immunophenotyping strategy was a subset of T cells marked by the specific expression of the small intestine-homing chemokine receptor CCR9, as well as increased expression of a number of other classical homing markers, such as *ITGA4* (CD49d) and *ITGAE* (CD103) (cluster 10; Fig. 6a and Additional file 4: Table S3). Analysis of surface-expressed markers identified an increased expression of CD38 in this cluster, along with the co-expression of *IL23R*, *IL12RB1* and *KLRB1* (CD161; Fig. 6a). Recently, a subset of CD38^+^CD62L^−^ effector T cells expressing gut-homing receptors, including CCR9, has been described in the blood in humans [30]. These cells were shown to have strong immunomodulatory properties mediated by the expression of T cell inhibitory receptors such as TIGIT, and their frequency was shown to be decreased in the blood of IBD patients [30]. In agreement with this putative regulatory function, our data confirmed the increased expression not only of *TIGIT*, but also of the T cell immune checkpoint molecules *ICOS*, *HAVCR2* (TIM-3) and *LAG3* (Fig. 6a). Consistent with their effector T cell phenotype, this cluster of CCR9^+^ T cells displayed increased expression of several classical genes associated with T cell effector function, including *FAS*, *ANXA5*, *CASP5* and *SELPG* (Fig. 6a). Furthermore, the pseudotime analysis demonstrated that these cells correspond to a highly differentiated cell state, located within the Treg and Th17 differentiation trajectories (Fig. 3a).

Of the 75 cells assigned to cluster 10, 36 (48.0%) were originally sorted from the CD127^low^CD25^low^ gate, while 37 (49.3%) were sorted from the CD127^low^CD25^hi^ gate. These data further support the differentiated state of this T cell subset and indicate that the sorting strategy employed in this study, strongly enriching for the frequency of cells in these two gates, allowed for the robust detection of this rare subset, which may have been missed in a more heterogeneous total CD4^+^ T cell population. In addition, these data also suggest that CCR9^+^ T cells display intermediate to elevated levels of IL-2RA (CD25) expression, consistent with their frequent detection within the conventional CD127^low^CD25^hi^ Treg gate. To investigate the putative regulatory function of these cells, we then compared the transcriptional profile of these cells with the identified memory Teff clusters (clusters 2, 5, 7, 8 and 9; Fig. 6b) or with the memory Treg clusters (clusters 3 and 4; Fig. 6c). In both comparisons, we observed a systematic upregulation of similar sets of genes in the CCR9^+^ T cells (cluster 10), consistent with the highly differentiated state of this cluster, and putative immunomodulatory properties. We did not observe a distinct upregulation of a set of Treg signature genes, suggesting that these cells do not represent a bona fide Treg subset. In addition, we observed a modest increased expression of FOXP3, when compared to memory Teffs, but lower than memory Tregs (Fig. 6a). As FOXP3 expression is known to be an imperfect marker of Tregs in humans, this intermediate expression could therefore represent transient upregulation in this subset of activated T cells. Moreover, despite the expression of several surface markers usually associated with Th17 cells, we observed no evidence for the expression of RORC (RORγt; Fig. 6a). These data suggest that CCR9^+^ T cells may respond to the same signalling and migration cues as Th17 cells, for example, IL-23, which could be responsible for their co-localisation and potential regulatory interaction in mucosal sites.

One distinguishing feature of this subset was the expression of the transcription factor *POU2AF1* (Fig. 6a). Although *POU2AF1* (encoding OCA-B) has been mainly characterised as a B cell-specific transcription factor in the blood, where it plays a role in B cell maturation [31], it has also been recently shown in mice to regulate the maintenance of memory phenotype and function in previously activated CD4^+^ T cells [32] and the differentiation of T follicular helper (Tfh) cells in the tissue [33].

### Single-cell comparison of mRNA and protein expression levels reveals modest and variable levels of correlation in primary CD4^+^ T cells

**Fig. 7:**
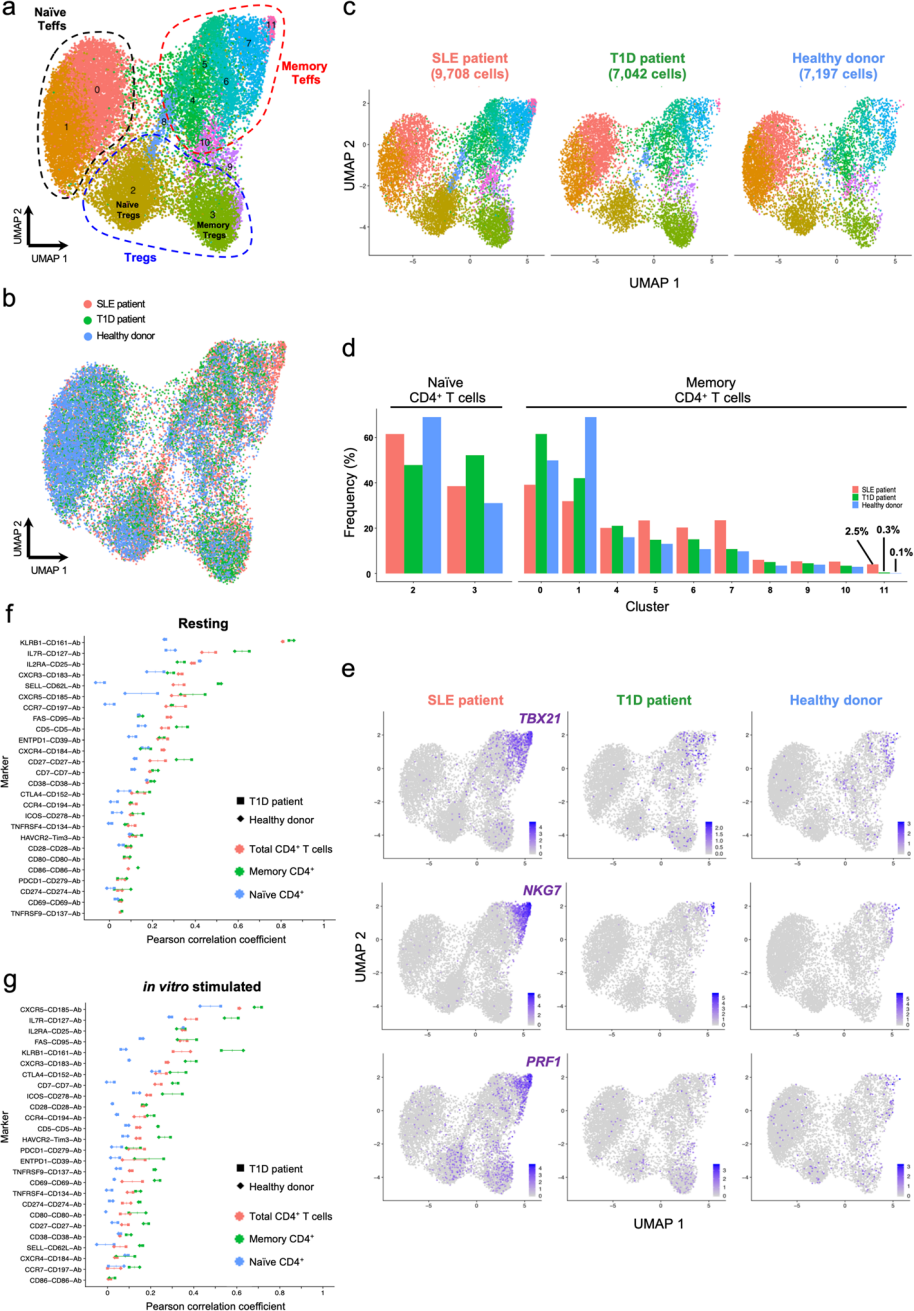
Data from independent experiments can be robustly integrated. **a** UMAP plot depicting the clustering of resting primary CD4^+^ T cells from one systemic lupus erythematosus (SLE; *n* = 9708 cells) patient, one type 1 diabetes (T1D; *n* = 7042 cells) patient and one healthy donor (*n* = 7197 cells). Data were integrated from two independent experiments using the same CD4^+^ T cell FACS sorting strategy (described in Fig. 1a). **b** Alignment of the integrated targeted transcriptomics and proteomics data generated from the three assessed donors in two independent experiments. **c** UMAP plots depicting the donor-specific clustering of the CD4^+^ T cells. **d** Relative proportion of the identified CD4^+^ T cell clusters in each donor. Frequencies were normalised to either the annotated naive or memory compartments to ensure higher functional uniformity of the assessed T cell subsets and to avoid alterations associated with the declining frequency of naive cells with age. **e** UMAP plots depicting the relative expression of the canonical Th1 transcription factor *TBX21* (encoding TBET) and the effector cytokines *NKG7* and *PRF1* on the three assessed donors. **f**, **g** Correlation (Pearson correlation coefficient) between mRNA and protein expression for 26 markers with concurrent mRNA and protein expression data in resting (**f**) and in vitro-stimulated (**g**) CD4^+^ T cells. The correlation was calculated in the total CD4^+^ T cells (red) or in the CD45RA^−^ memory (green) or CD45RA^+^ naive (blue) T cell subsets separately. Individual-level correlation in the type 1 diabetes (T1D) patient (square) and healthy donor (diamond) and median correlation in both donors are displayed in the figure

Given that the main advantage of this combined targeted scRNA-seq and proteomic approach is the ability to immunophenotype large numbers of cells from multiple donors, we next investigated whether we were able to integrate data generated from independent experiments. We replicated the initial experiment using the same pre-sorting strategy to isolate the three assessed CD4^+^ T cell subsets from an individual with type 1 diabetes and one healthy donor. To further test the potential of the protein quantification, we extended the AbSeq panel to 43 protein targets expressed on CD4^+^ T cells (Additional file 1: Table S1). In agreement with the initial experiment, unsupervised clustering of the 23,947 cells passing QC revealed a similar discrimination of CD4^+^ T cell subsets (Fig. 7a) and good alignment of the data from the three donors (Fig. 7b). Analysis of the donor-specific distribution of the identified CD4^+^ T cell clusters showed that the frequency of the putative CD4^+^ cytotoxic Th1 subset (cluster 11), marked by the co-expression of TBET and effector-type cytokines, was highly increased in the SLE patient (Fig. 7c–e). To avoid age-specific differences in the relative distribution of the CD45RA^+^ naive and CD45RO^+^ memory compartments in these donors, we normalised the analysis to the memory T cell clusters only, which we were able to robustly annotate using the AbSeq data for the expression of CD45RA and CD45RO. Although we detected a few cells with this activated Th1 phenotype in circulation from every donor, there was a very substantial expansion in the SLE patient (2.5% of memory CD4^+^ T cells compared to 0.3% and 0.1% in the T1D patient and healthy donor, respectively; Fig. 7d), suggesting that it could represent a pathogenic CD4^+^ T cell subset associated with systemic autoimmunity in this patient. In addition, we also integrated the full dataset of resting and in vitro-stimulated CD4^+^ T cells from the three donors, resulting in a combined dataset of 43,656 cells. Despite the known activation-induced changes in the transcriptional profile of in vitro-stimulated T cells—including cytokine expression—we observed a good integration of the datasets, yielding similar annotation of the resulting T cell clusters (Additional file 3: Figure S8 and Additional file 5: Table S4). The only exception was the separation of the in vitro-activated naive T cells into a distinct cluster (cluster 3), caused by the upregulation of cytokine expression (most notably IL-2), which was completely absent in resting cells. In contrast, we observed a more consistent integration of the memory clusters, and particularly the memory Teff cells, thereby providing additional information and increased cell numbers for the functional annotation of this dataset.

The parallel quantification of mRNA and protein expression for a large number of genes expressed in CD4^+^ T cells in this experiment provided a unique opportunity to investigate their systematic correlation at the single-cell level. From the 43 proteins quantified with AbSeq, 26 were also assessed at the transcriptional level and detected in our CD4^+^ T cell dataset. Generally, we observed relatively weak (mean Pearson correlation coefficient = 0.214) but variable levels of correlation in total resting CD4^+^ T cells, ranging from 0.049 for *TNFRSF9* to 0.808 in *KLRB1* (encoding CD161; Fig. 7). Furthermore, we note that with the exception of *CXCR5*, the estimated correlations were very consistent between the two independent donors (Fig. 7f). These findings were consistent with previous observations [9, 10] and suggest that primary CD4^+^ T cells are highly specialised cells, where transcription is subject to tight regulation to avoid excessive energy consumption by the cell and to control effector function. As expected, by normalising our analysis to a functionally more homogeneous population of memory CD4^+^ T cells, we observed higher levels of correlation (mean = 0.233), which is consistent with their increased expression of the majority of the assessed T cell markers. A slightly decreased correlation (mean = 0.178) was observed in in vitro-stimulated CD4^+^ T cells (Fig. 7g).

### Parallel mRNA and protein profiling provides increased cell-type resolution of the heterogeneous CD45^+^ immune cell population in blood and tissue

**Fig. 8:**
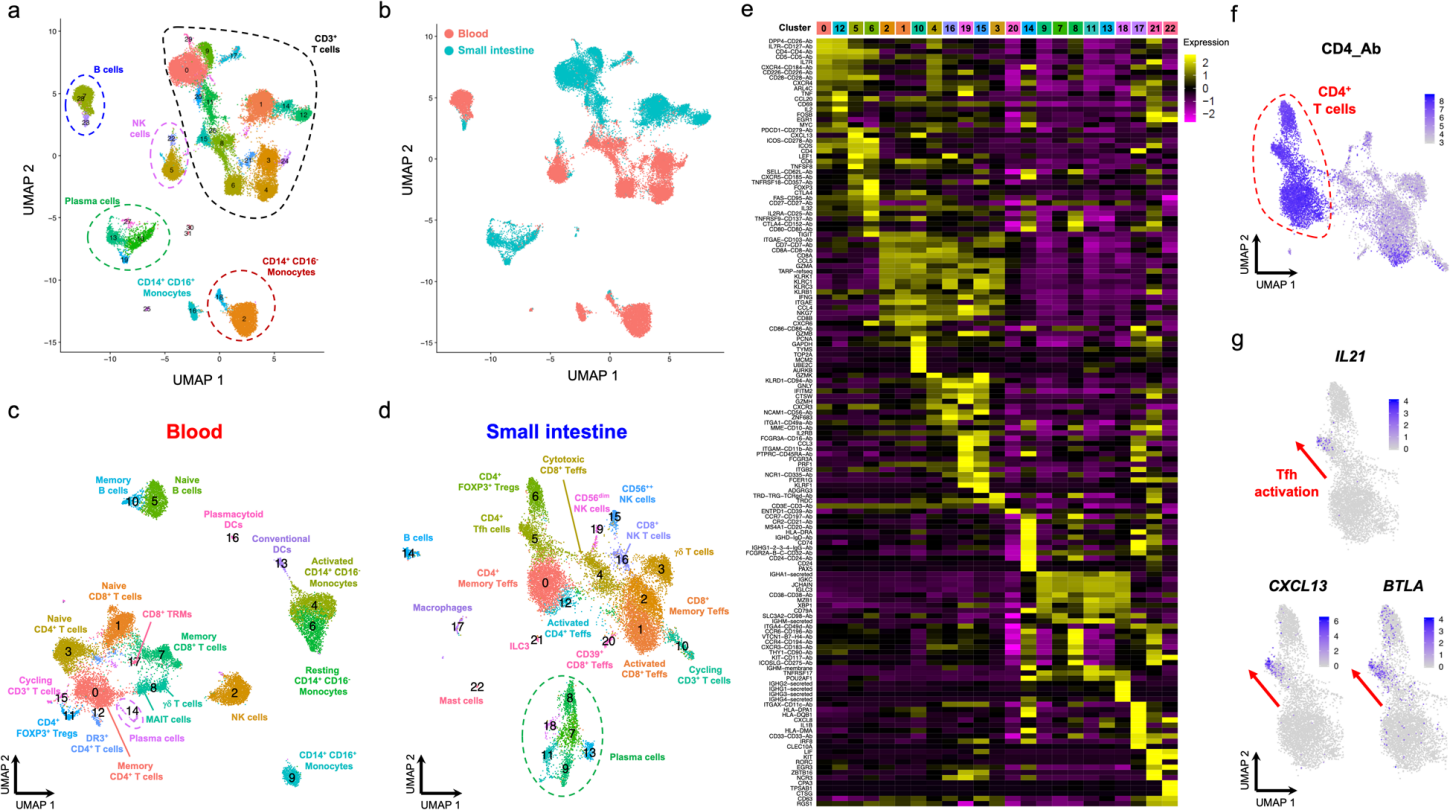
Targeted scRNA-seq and proteomics approach delineates distinct functional subsets in a heterogeneous population of CD45^+^ immune cells isolated from blood and tissue. **a** UMAP plot depicting the clustering of the targeted scRNA-seq and transcriptional data of a heterogeneous population of total CD45^+^ cells (*n* = 31,907) isolated from blood and a paired duodenal biopsy from two coeliac disease (CD) patients with active disease. **b** Sample Tag information identifies samples isolated from the blood (red) or from the paired duodenal biopsy (teal). **c**, **d** UMAP plot depicting the clustering of the CD45^+^ cells isolated from the blood (**c**) or the paired duodenal biopsy (**d**). **e** Heatmap displaying the top 10 differentially expressed genes in each identified cluster from the CD45^+^ immune cells isolated from the duodenal biopsies. **f** UMAP plot depicting the expression of CD4 at the protein level (AbSeq) within the CD3^+^ T cells isolated from the small intestine. **g** Gradient of expression of the Tfh effector genes *IL21*, *CXCL13* and *BTLA* in tissue-resident CD4^+^ T cells. DR3, death-receptor 3 (encoded by *TNFRSF25*); TRM, tissue-resident memory T cells; MAIT, mucosal-associated invariant T cells; ILC3, type 3 innate lymphoid cell

To investigate how this targeted scRNA-seq and transcriptomics approach performed on a more heterogeneous population of immune cells, we isolated total CD45^+^ cells from the blood and a matching duodenal biopsy from two coeliac disease (CD) patients with active disease. In this experiment, we captured 31,907 single cells that passed QC and expanded the AbSeq panel to the detection of 68 protein targets (Additional file 1: Table S1). As expected, we observed a very defined clustering of the different populations representing the CD45^+^ immune cells (Fig. 8a). Consistent with previous data [34, 35], we found a clear separation of cells isolated from either blood or the small intestine (Fig. 8b), indicating a transcriptional signature of tissue residency. Furthermore, clustering of cells isolated from blood (Fig. 8c and Additional file 3: Figure S9a) or tissue (Fig. 8d) separately revealed the expected cell populations. The main distinction was the relative distribution of the immune populations, with a marked increased representation of B cell, NK cell and CD14^+^CD16^−^ monocyte populations in the blood, and a significantly increased proportion of plasma cells in the small intestine. In agreement with our findings in CD4^+^ T cells, we found that the acquisition of a memory phenotype was the main driver of the clustering of both CD4^+^ and CD8^+^ T cells (Additional file 3: Figure S9b,c). In addition, we identified other clusters of non-conventional T cells, including a subset of γδ T cells and mucosal-associated invariant T cells (MAIT) in the blood, which shared similarities with the transcriptional signature of memory CD8^+^ T cells, marked by the expression of effector-type cytokines genes, such as *NKG7* (Additional file 3: Figure S9c). In contrast, tissue-resident CD4^+^ T cells isolated from the small intestine were restricted to the memory phenotype and displayed a markedly different subset distribution, including a substantially enlarged population of FOXP3^+^ Tregs (Fig. 8e, f). Moreover, the simultaneous assessment of the protein expression of CXCR5, ICOS and PD-1 identified a cluster of Tfh cells (Fig. 8e), which could be distinctly clustered along a trajectory of Tfh cell activation, as illustrated by the gradient of expression of key Tfh functional transcripts, such as *IL21*, *CXCL13* and *BTLA* (Fig. 8g).

Similarly, we also identified distinct trajectories of cell differentiation in other immune cell types, as illustrated by the gradient of differentiation and class switching of B cells in the blood (Additional file 3: Figure S10a-c). Peripheral B cells were clearly dominated by a naive IgD^+^IgM^+^ CD27^−^ subset, and only a small fraction of class-switched IgG^+^ CD27^+^ memory B cells, which was consistent with the young age of the CD patients. In contrast, tissue-resident B cells were much less abundant and contained mostly cells with a class-switched IgG^+^ CD27^+^ memory phenotype. In addition, we identified a vastly expanded population of antibody-secreting plasma cells (Additional file 3: Figure S10d,e). Of note, because we were able to specifically assess the expression of the secreted Ig isotypes, we could discriminate the different functional plasma cell subsets, including a very abundant population of IgA-secreting plasma cells (Additional file 3: Figure S10f), which are known to play a critical role in the interaction with the microbiome in the gut. Together, these data provide an example of the power of this multi-omics approach to identify trajectories of cell differentiation and cell states in diverse immune cell and tissue types.

## Discussion

The advent of scRNA-seq has proved to be transformative in shaping our understanding of the complexity and function of the human immune system [36, 37]. However, currently, both the elevated costs to perform these experiments and the reliance on transcriptional data alone pose significant challenges to the widespread practical applicability of these technologies. In this study, we present an integrated, cost-effective approach to sensitively assess the simultaneous expression of mRNA and protein for hundreds of key immune targets at the single-cell level using the AbSeq technology.

Recently, two similar approaches, CITE-seq [9] and REAP-seq [10], have been described to measure protein expression using oligo-conjugated antibodies in parallel with scRNA-seq data. Furthermore, other applications are currently being developed to integrate the growing portfolio of single-cell omics technologies [38, 39]. A fundamental difference with the approach described in this study is that these technologies all rely on whole-transcriptome data, providing a high-level cross-sectional representation of all polyA mRNA transcripts in the cell. In contrast, by using targeted scRNA-seq, we are relying on prior knowledge to specifically assess the expression of hundreds of selected genes in single cells. Moreover, we show here that a targeted approach provides sensitive quantification of the selected genes at a fraction - approximately 1/10th - of the cost , as it avoids the detection of highly expressed invariant housekeeping genes, which take up the vast majority of the whole-transcriptome scRNA-seq libraries. This reduced sequencing requirement for targeted scRNA-seq allows to allocate a larger proportion of the sequencing resources to the protein library, resulting in a 3- to 4-fold reduced cost per cell—assuming similar coverage for protein quantification—compared to whole-transcriptome scRNA-seq (summarised in Additional file 6: Table S5). The features and cost considerations are critical to inform on the optimal use of a targeted or whole-transcriptome approach. The increased sensitivity of a targeted approach is particularly relevant for the accurate assessment of lowly expressed genes with critical regulatory function, such as transcription factors, which are often poorly quantified using traditional whole-transcriptome scRNA-seq data. It therefore provides a knowledge-based approach to validate and extend whole-transcriptome scRNA-seq findings, which can be widely implemented in any research or clinical setting. Similar to other widely implemented knowledge-based single-cell immunophenotyping tools such as flow cytometry and CyTOF, the highly customisable nature of this approach is critical to investigate specific research questions with very high sensitivity and in a larger number of samples. However, in contrast to CyTOF, which is inherently time-consuming and requires the availability of large numbers of cells to maximise the information generated by each run, this technology is ideally suited for unique and highly valuable clinical samples, for which cell availability and number are major practical constraints. Furthermore, the digital nature and lack of spectral overlap issues with the AbSeq measurements can mitigate some of the limitations associated with flow cytometry, allowing accurate quantification of zero or very low copy numbers, which are usually difficult to discriminate by flow cytometry, and could reveal meaningful biological differences.

The increased sensitivity and the high number of parameters simultaneously assessed using this multi-omics approach can also lead to unanticipated novel biological findings. An illustrative example of this potential is the identification of the APC-restricted B7 family molecules CD80 and CD86 co-stimulatory proteins as markers of activated Tregs in peripheral blood. Despite their predominant function in APCs, endogenous expression of CD80 [26, 27] and CD86 [28] has been previously demonstrated on activated T cells in humans. Time-course analysis of the expression of these molecules in an in vitro activation model revealed the co-localisation of these molecules with CD28 on the surface of activated T cells [40]. Our data demonstrate that the expression of CD80 and CD86 can also be detected in humans on the surface of primary T cells in blood, predominantly in Tregs. Notably, our results also reveal that induction of IL-2 signalling was sufficient for the maintenance of B7 protein expression on activated T cells, therefore providing a rationale for the observed AbSeq findings, showing restricted expression of these molecules ex vivo, particularly CD86, on the surface of a subset of activated mTregs, which are known to display the highest sensitivity for IL-2 signalling. Consistent with these observations, CD86 expression in T cells has been previously shown to be critically dependent on IL-2 signalling [41]. Taken together, these data provide further evidence pointing to a functional role of B7 molecules in the regulation of a T cell response and suggest an intriguing potential uncharacterised role of CD86 in Treg function.

In contrast to CD86 protein expression which is often associated with high levels of intracellular CTLA-4 (Fig. 4e), CD80 protein expression could also be detected on Tregs with lower levels of CTLA-4 (Fig. S7c) and displayed a broader expression profile in other activated T cell subsets. In particular, the pseudotime analysis performed in our dataset identified CD80 as a marker of the temporal differentiation of Th17 cells (Fig. 3d), which may provide a mechanistic rationale for the recently reported suppression of Th17 differentiation in response to anti-CD80 treatment in mice [42]. Furthermore, we also note a distinct co-expression of CD80 protein and HLA class II mRNA (*HLA-DRA*) in a subset of activated Th1 cells (Fig. 2d), which could indicate recent activation in the context of strong TCR signalling required to induce the differentiation of Th1 cells [43, 44].

Currently, we cannot rule out that CD80/CD86 molecules detected on Tregs could be acquired exogenously through mechanisms such as CTLA-4-mediated trans-endocytosis [45, 46] or TCR-mediated trogocytosis [47–49]. Although these models are consistent with the virtual lack of mRNA detection in our ex vivo T cell datasets and with the observed co-expression with CTLA-4, it is unlikely that such mechanisms could lead to long-lasting protein expression on the surface of T cells. In agreement with endogenous production accounting for the majority of CD80/CD86 expression on T cells, exogenous acquisition of B7 molecules from APCs has been shown to be a rapid process that immediately follows T cell activation [50, 51] and could therefore only account for transient surface expression. In contrast, endogenous expression of B7 family molecules by T cells has been shown to be a late-stage and more stable activation marker, which is more consistent with the broader pattern of expression observed in this study. Interestingly, a recent study suggested a novel role for B7 molecules in regulating CD8^+^ T cell population dynamics, by controlling T cell expansion through T-T cell signalling via CD28 and CTLA-4 [29]. These findings suggest a major function of B7 family molecules in T cell biology, providing critical regulatory signals that curtail chronic T cell activation, which could be particularly relevant in the context of high T cell density in inflammatory environments. Consistently, ablation of B7/CD28 signalling in CD80/CD86 knock-out mice [52] or clinically with CTLA-4Ig (abatacept) has been shown to impair T cell regulation and lead to aggressive secondary autoimmunity [53, 54].

Another example of the potential of this multi-omics approach to reveal novel biological findings was the identification of a rare subset of highly differentiated T cells marked by the expression of the small intestine-homing marker CCR9 (cluster 10). Detailed characterisation of their cell surface and transcriptional profile in vivo revealed specific expression of *CD38* and a set of immune checkpoint molecules: TIGIT, ICOS, CTLA-4 and LAG-3. This phenotype is consistent with a recently described subset of gut-homing CD38^+^TIGIT^+^CD62L^−^ effector T cells with a putative immunoregulatory role in IBD [30]. In contrast to Joosse et al., our multiparametric characterisation of the CCR9^+^ T cells at the single-cell level resulted in a much more restricted T cell subset compared to the more frequent CD38^+^TIGIT^+^CD62L^−^ T cell population described by flow cytometry in their study. Nevertheless, given the very specific expression of CCR9 in cluster 10 that we show in this study, it is plausible that an enrichment of this subset amongst the more heterogeneous CD38^+^TIGIT^+^CD62L^−^ T cell population could be responsible for the reported gene expression signatures. In addition to CD38, another feature of the CCR9^+^ T cell subset was the increased expression of *IL23R* and *IL12RB1*, which encode for the two subunits of the IL-23 receptor. The clinical relevance of the IL-12/IL-23 signalling pathway to gut inflammatory disease has been well established [55], and a coding variant in the IL-23R gene has been strongly associated with the susceptibility to inflammatory bowel disease (IBD) [56]. Together, these data point to a role of this subset of CCR9^+^ T cells in regulating pro-inflammatory Th17 immunity in response to IL-23 signalling in the gut, thereby preventing chronic inflammation, and provide new tools to monitor their frequency in the blood in the context of gut inflammatory conditions.

An important finding from this study and other related studies [9, 10] is the generally low levels of correlation between mRNA and protein expression in primary CD4^+^ T cells at the single-cell level. One possible explanation for this observation is that reduced sensitivity of scRNA-seq to quantify mRNA expression may be leading to an underestimation of the correlation coefficients. However, we note that there are notable exceptions, such as CD161, which displayed a high correlation between mRNA and protein levels at 0.847 in memory CD4^+^ T cells, demonstrating that a systematic error in the quantification of mRNA levels by scRNA-seq technologies is not the only factor contributing to the observed low level of correlation. These findings therefore underscore the importance of parallel protein quantification to better identify stable cellular phenotypes associated with cell function. In contrast to mRNA expression, proteins display a much larger dynamic range of expression and longer half-life [57, 58], resulting in much higher copy numbers and more accurate and reliable quantification compared to their mRNA counterparts. This is particularly relevant in differentiated primary cells, such as CD4^+^ T cells, where transcription is tightly regulated to maintain effector function. These low copy numbers result in increased stochastic variation in mRNA quantification and dropout rate, which impair the accuracy of single-cell methods that rely only on transcriptional data. Furthermore, mRNA profiling provides only a snapshot of the current functional state of the cell, which can be better assessed with combined protein expression data. An illustration of the power of this multi-omics approach is the detailed trajectories of differentiation that we identified in resting primary CD4^+^ T cells, which were recapitulated by precise gradients of mRNA expression. The sensitivities of these measurements combined with the high numbers of cells analysed lend themselves to identify gradual and subtle changes in cell states, which are critical to identify dynamic changes reflecting mechanisms of functional adaptation in a heterogeneous cell population.

## Conclusions

In this study, we show that combined targeted scRNA-seq and protein expression analysis provides a high-resolution map of the human immune cells in blood and tissue and reveals novel biological insights into the biology of CD4^+^ T cells, as illustrated by the identification of CD80/CD86 expression on activated Tregs and a rare CCR9^+^ T cell subset in the blood, with tissue-homing properties and putative immunomodulatory function. Our data provide a proof-of-principle for the implementation of this integrated approach as a widely applicable and cost-efficient research tool for immunologists. This approach could be particularly valuable in a clinical setting for the characterisation of rare patient samples with limited cell numbers, as well as to assess the functional consequence, at the single-cell level, of targeting key biological pathways in vivo, such as in patients treated with immunotherapeutic drugs.

## Supplementary information

**Additional file 1: Table S1.** FACS and AbSeq anti-human monoclonal antibodies used in this study.

**Additional file 2: Table S2.** Summary of the cell capture efficiency and multiplet rates for the experiments performed in this study.

**Additional file 3: Figure S1.** Protein expression displays much larger dynamic range of expression. **Figure S2.** Targeted scRNA-seq provides increased sensitivity to detect lowly expressed transcripts. **Figure S3.** Effects of different data normalisation methods on cell clustering. **Figure S4.** Differential expression in the identified resting and in vitro stimulated primary CD4^+^ T-cell subsets. **Figure S5.** In vitro stimulation reinforces the trajectories of CD4^+^ T cell differentiation. **Figure S6.** Interplay between the BACH2 and BLIMP-1 transcriptional programmes regulates CD4^+^ Treg differentiation in humans. **Figure S7.** Characterising the expression of the B7 molecules CD80 and CD86 on CD4^+^ T cells. **Figure S8.** Integration of data from resting and in vitro stimulated CD4^+^ T cells. **Figure S9.** Single-cell mRNA and protein quantification identifies distinct functional populations of human circulating CD3^+^ T cells. **Figure S10.** Targeted multi-omics approach reveals trajectories of B-cell differentiation and class switching in blood and tissue.

**Additional file 4: Table S3.** Summary statistics of the differentially-expressed markers (protein and mRNA targets) in the CCR9^+^ T-cell cluster 10.

**Additional file 5: Table S4.** Summary statistics of the differentially-expressed markers in the combined resting and in vitro stimulated CD4^+^ T-cell dataset.

**Additional file 6: Table S5.** Cost comparison of targeted and whole-transcriptome scRNA-seq systems.

## Data Availability

All scRNA-seq data generated in this study are available from the NCBI’s Gene Expression Omnibus (GEO), under accession number GSE150060 [59].

## Abbreviations

APCs: Antigen-presenting cells
CyTOF: Cytometry by time of flight
PBMCs: Peripheral blood mononuclear cells
CD: Coeliac disease
FACS: Fluorescence-activated cell sorting
FC: Fold change
IBD: Inflammatory bowel disease
scRNA-seq: Single-cell RNA sequencing
SLE: Systemic lupus erythematosus
T1D: Type 1 diabetes
TCR: T cell receptor
Teff: Effector T cell
Tfh: Follicular helper T cell
Treg: CD4^+^ regulatory T cell
UMAP: Uniform Manifold Approximation and Projection
UMI: Unique molecular identifier
