# Supplementary material for: Discovery of CD80 and CD86 as recent activation markers on regulatory T cells by protein-RNA single-cell analysis
Source: Genome Med. 2020 Jun 24;12:55. doi: 10.1186/s13073-020-00756-z (PMC7315544; doi:10.1186/s13073-020-00756-z)
Supplement: Supplementary file 3 — Additional file 3: Figure S1. Protein expression displays much larger dynamic range of expression. Figure S2. Targeted scRNA-seq provides increased sensitivity to detect lowly expressed transcripts. Figure S3. Effects of different data normalisation methods on cell clustering. Figure S4. Differential expression in the identified resting and in vitro stimulated primary CD4+ T-cell subsets. Figure S5. In vitro stimulation reinforces the trajectories of CD4+ T cell differentiation. Figure S6. Interplay between the BACH2 and BLIMP-1 transcriptional programmes regulates CD4+ Treg differentiation in humans. Figure S7. Characterising the expression of the B7 molecules CD80 and CD86 on CD4+ T cells. Figure S8. Integration of data from resting and in vitro stimulated CD4+ T cells. Figure S9. Single-cell mRNA and protein quantification identifies distinct functional populations of human circulating CD3+ T cells. Figure S10. Targeted multi-omics approach reveals trajectories of B-cell differentiation and class switching in blood and tissue. [file 13073_2020_756_MOESM3_ESM.docx]

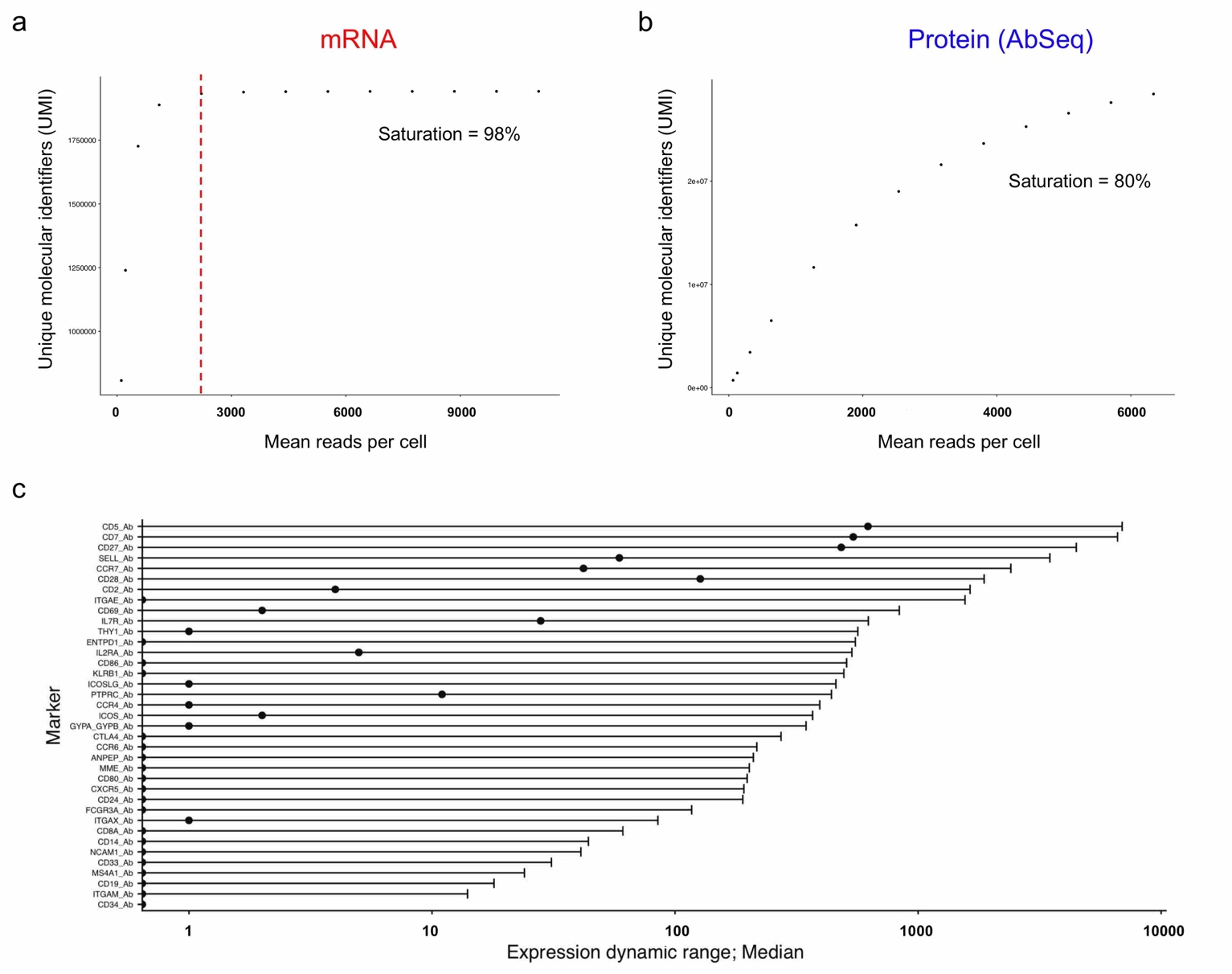


Fig S1. Protein expression displays much larger dynamic range of expression.

(**a, b**) Sequencing saturation metrics of the mRNA (**a**) and protein (**b**) libraries. Saturation of the sequencing libraries was quantified as the number of identified distinct, non-clonally amplified cDNA molecules, marked by a unique molecular identifier (UMI), with increasing sequencing coverage. (**c**) Data shown depicts the distribution (median and range) of the expression of the 42 protein targets measured by AbSeq. Data was derived from the analysis of the first experiment performed on pre-sorted resting CD4^+^ T cell populations from a systemic lupus erythematosus (SLE) patient.

Fig S2. Targeted scRNA-seq provides increased sensitivity to detect lowly expressed transcripts.

(**a, b**) Gating strategy for the delineation of equivalent CD4^+^ regulatory T cell (Treg) populations using protein expression from oligo-conjugated antibodies (as outlined in **Fig. 1b**). Data was obtained from two whole-transcriptome scRNA-seq datasets combining mRNA and protein expression data (available at <https://support.10xgenomics.com/single-cell-gene-expression/datasets>). mRNA libraries were generated using either the 10X Genomics v3 (**a**) or NextGEM (**b**) chemistries. (**c**) Frequency of FOXP3^+^ cells (defined as cells expressing >= 1 *FOXP3* UMI) detected by each platform for CD4^+^ T cells identified in either the CD127^low^CD25^+^ (Treg) or Teff (non-Treg) gates. The sequencing coverages (defined as average number of reads per cell) of each experiment are shown in the figure. (**d**) Distribution of the *FOXP3* UMI counts detected in the three assessed experiments.

Fig S3. Effects of different data normalisation methods on cell clustering.

(**a, b**) UMAP plots depicting the clustering of the sorted resting CD4^+^ T cells (N = 9,708). Data corresponds to the UMAP plots shown in Fig. 1-3, and were obtained following two alternative data normalisation methods: (i) a hybrid approach scaling the expression of the protein (AbSeq) and mRNA libraries prior to clustering (**a**); and (ii) a transcriptomics approach using only the mRNA expression data (**b**). (**c,d**) Heatmaps depict the number of cell barcodes assigned to the annotated naïve Teff (black), Treg (blue), memory Teff (red) and CCR9^+^ T-cell (green) clusters when comparing the original and hybrid (**c**) or mRNA only (**d**) data normalisation methods. Clusters were annotated manually based on their respective protein and mRNA expression profiles to match the annotation in **Fig. 1d**. The cluster assignment consistency (represented as a percentage of the original assignment) is shown for the matching annotated clusters in the diagonal of each plot.


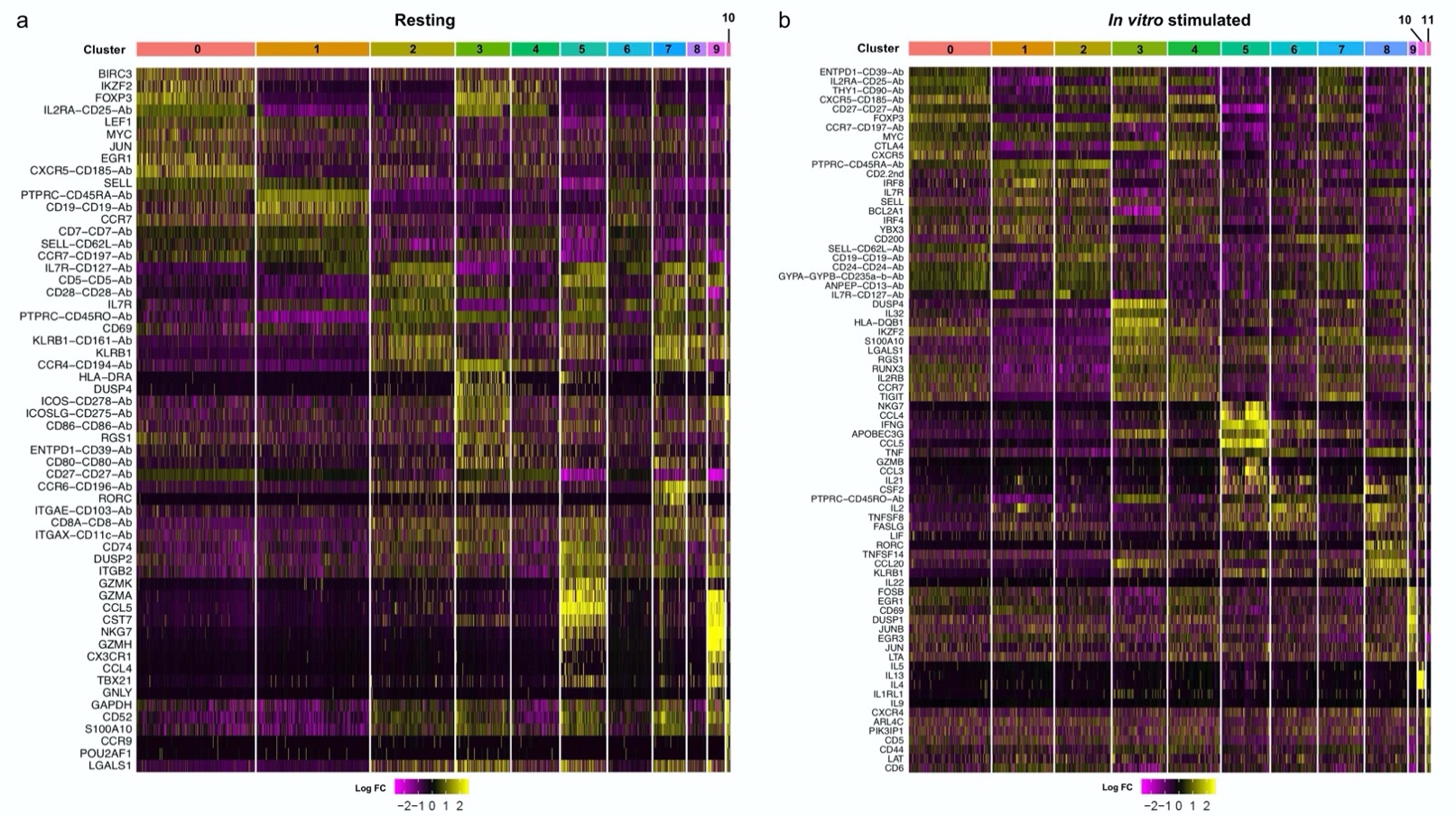


**Fig S4. Differential expression in the identified resting and *in vitro* stimulated primary CD4^+^ T-cell subsets.**

(**a, b**) Heatmaps displaying the top 10 differentially expressed genes in each identified resting (**a**) or *in vitro* stimulated (**b**) CD4^+^ T-cell clusters. Stimulation condition involved a short period of incubation (90 min) with PMA + ionomycin.


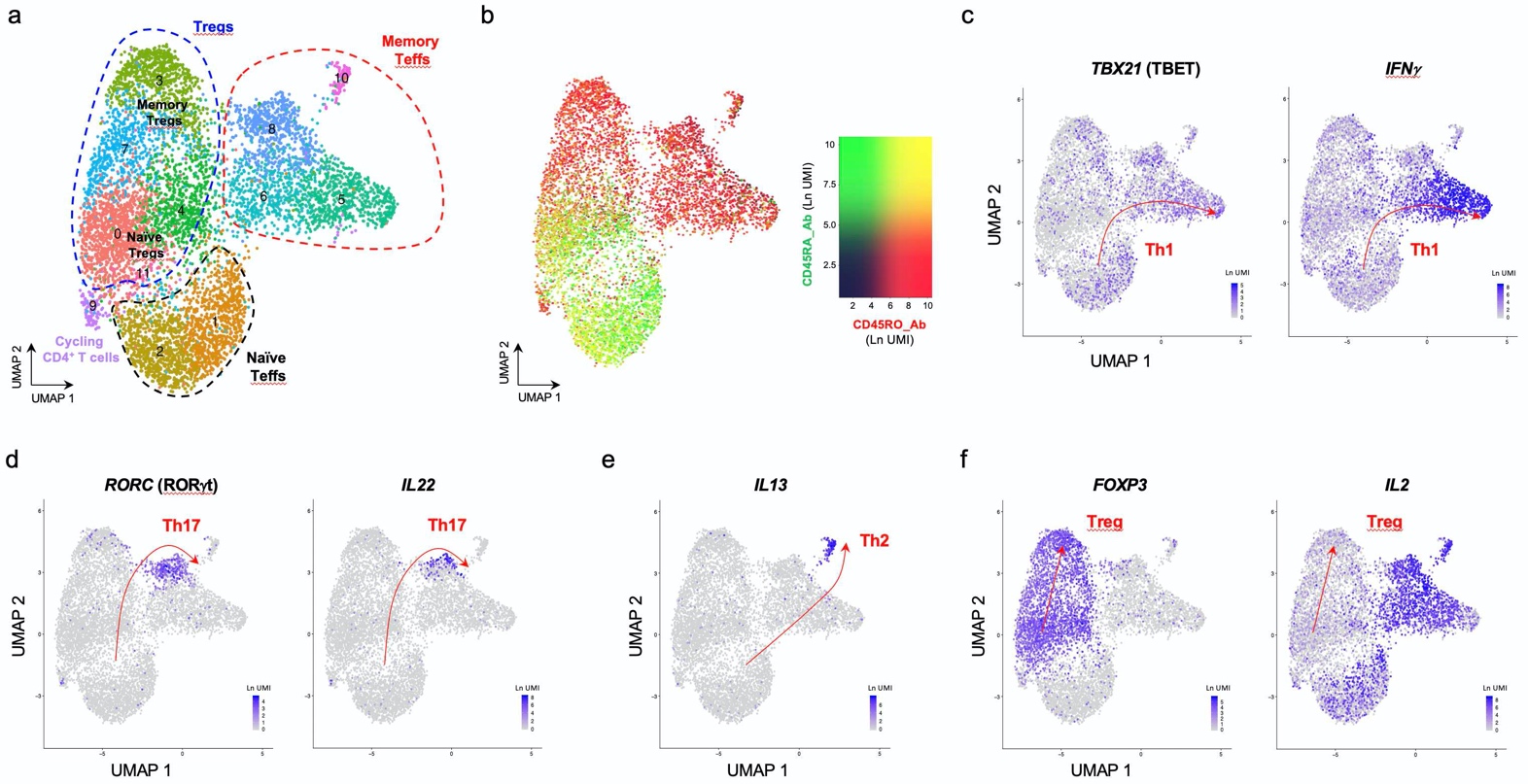


**Fig S5. *In vitro* stimulation reinforces the trajectories of CD4^+^ T cell differentiation.**

**(a)** UMAP plot depicting the clustering of the *in vitro* stimulated primary CD4^+^ T cells (n = 7,265) isolated from blood of a systemic lupus erythematosus (SLE) patient. Stimulation condition involved a short period of incubation (90 min) with PMA + ionomycin. (**b**) Data shown depicts the overlaid protein expression levels of the CD45RA (black to green) and CD45RO (black to red) isoforms in each CD4^+^ T cell following *in vitro* activation with PMA + ionomycin. (**c, d**) UMAP plots depicting the co-expression of the CD4^+^ T-cell lineage-defining CD4^+^ Th1 transcription factor TBET and the Th1 effector cytokine IFN-γ (**c**), as well as the Th17 transcription factor RORγt and the Th17 effector cytokine IL-22 (**d**) after *in vitro* stimulation with PMA + ionomycin for 90 min. (**e**) Expression of the canonical Th2 effector molecule IL-13 in *in vitro* stimulated CD4^+^ T cells. (**f**) UMAP plot depicting the expression of the Treg transcription factor FOXP3 and the prototypical CD4^+^ Th1 Teff cytokine IL-2 in the identified CD4^+^ T-cell subsets after *in vitro* stimulation with PMA + ionomycin for 90 min.


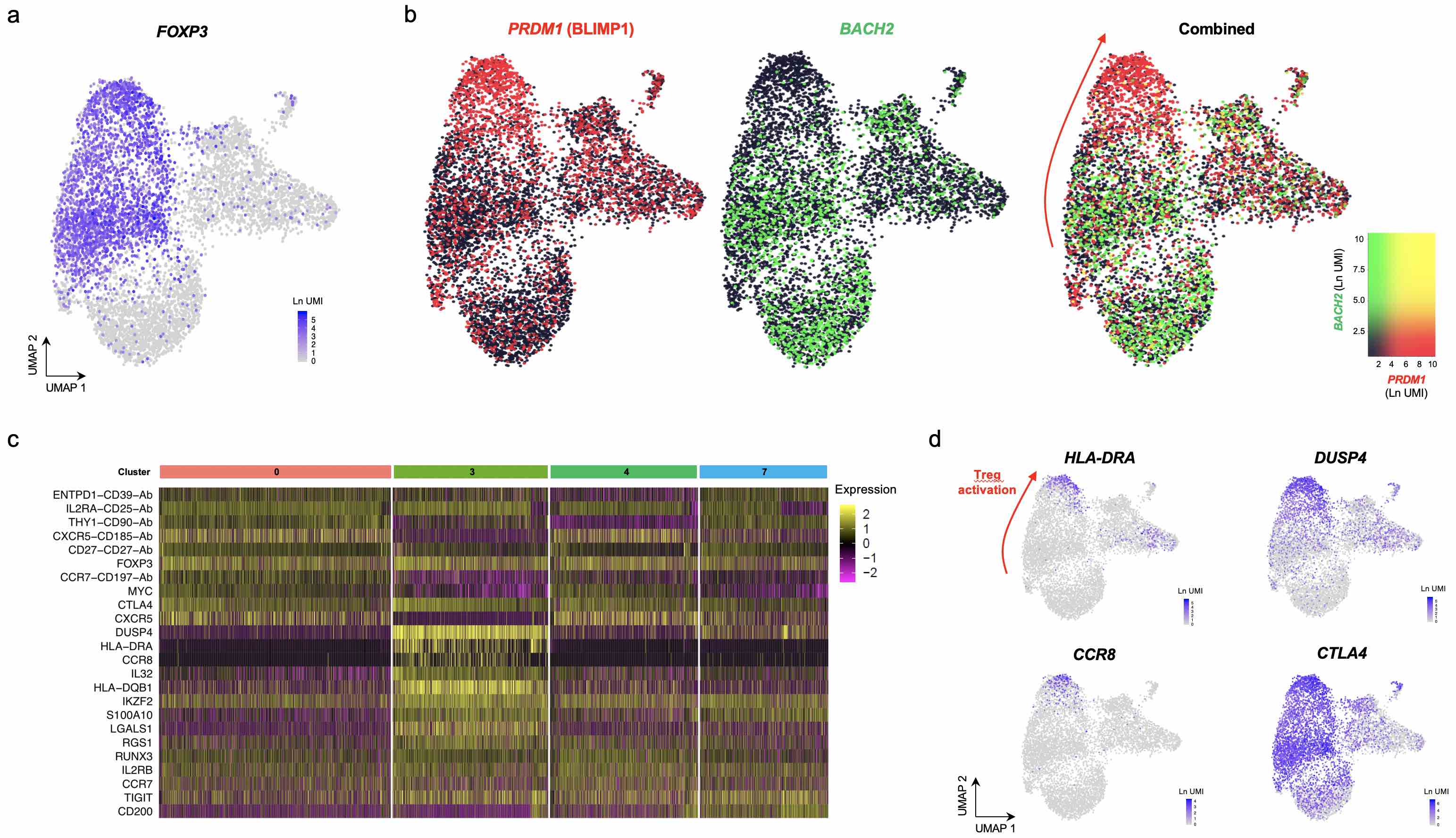


**Fig S6. Interplay between the BACH2 and BLIMP-1 transcriptional programmes regulate CD4^+^ Treg differentiation in humans.**

(**a**) UMAP plot depicting the expression of the canonical Treg transcription factor FOXP3 in the identified *in vitro* stimulated CD4^+^ T-cell clusters. (**b**) Data shown depicts the overlaid protein expression levels of the transcription factors BACH2 (black to green) and BLIMP-1 (encoded by *PRDM1*; black to red) in each CD4^+^ T cell following *in vitro* activation with PMA + ionomycin. (**c**) Heatmap displaying the top 10 differentially expressed genes within the four identified Treg clusters following *in vitro* stimulation with PMA + ionomycin. (**d**) UMAP plots depicting the gradient of expression of highly differentially expressed genes in the cluster of activated Tregs (cluster 3) following *in vitro* stimulation with PMA + ionomycin. Red arrows in this figure indicate the gradient of decreasing *BACH2* and concomitant gain in *PRDM1* expression associated with the gradual expression of Treg activation molecules and the acquisition of an activated Treg phenotype.


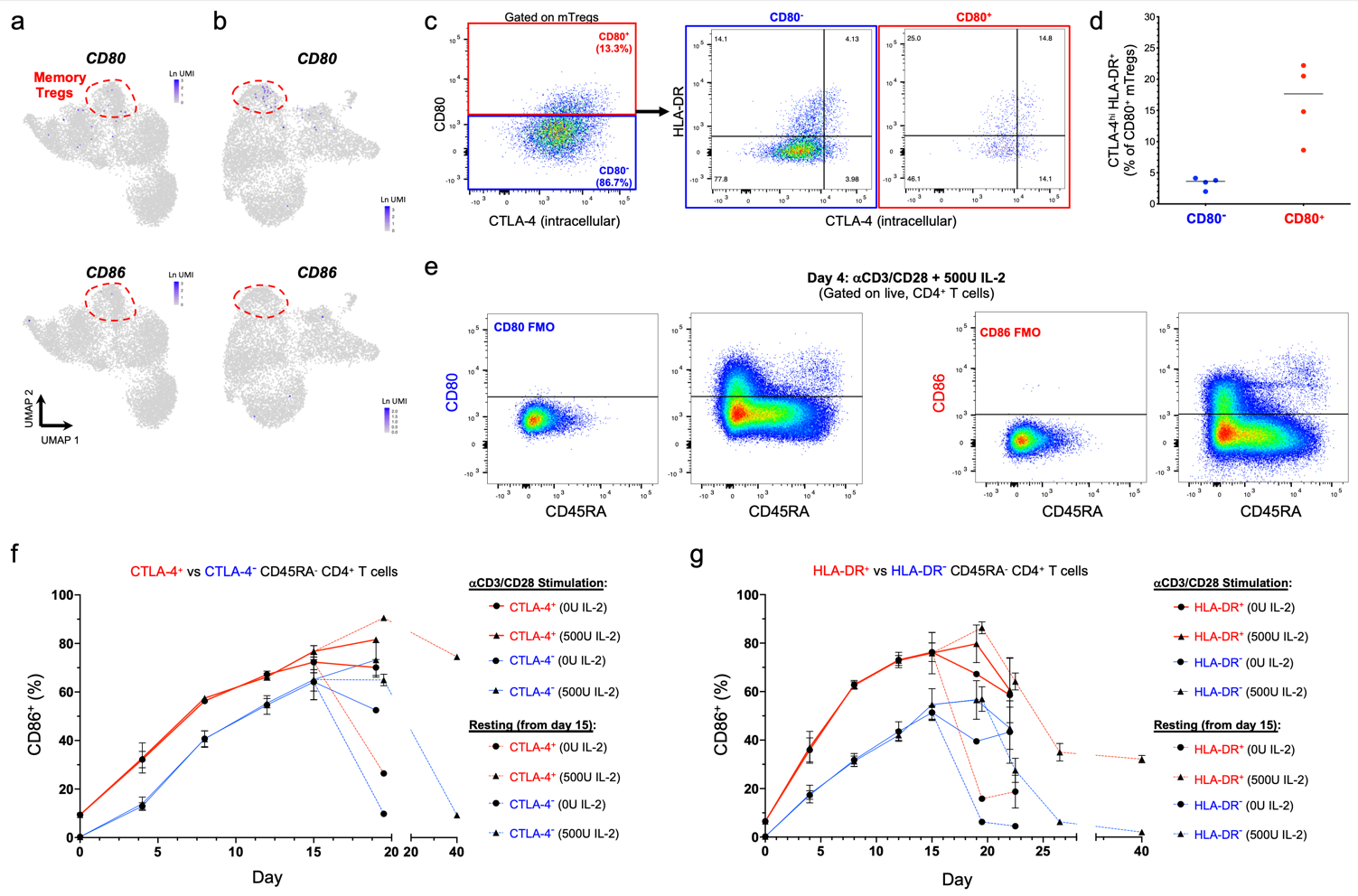


**Fig S7. Characterising the expression of the B7 molecules CD80 and CD86 on CD4^+^ T cells.**

(**a,b**) UMAP plots depicting the mRNA expression levels of the co-stimulatory molecules CD80 and CD86 in the identified CD4^+^ T-cell subsets in resting (**a**) and *in vitro* stimulated (**b**) conditions. Dashed lines delineate the identified activated Treg clusters. (**c**) Co-expression of CD80 and the CD4^+^ T-cell activation markers CTLA-4 and HLA-DR was assessed by flow cytometry in CD86^+^ (red) and CD86^–^ (blue) mTregs. (**d**) Scatter plots depict the frequency (median) of CTLA-4^hi^ HLA-DR^+^ cells within CD80^+^ and CD80^–^ mTregs. Expression of CTLA-4 was assessed by intracellular immunostaining. (**e**) Gating strategy for the delineation of CD80^+^ (blue) and CD86^+^ (red) cells in *in vitro* stimulated CD4^+^ T cells. Plot depicts an illustrative example obtained at day 4 post-stimulation for cells cultured with 500U IL-2. Background levels of CD80 and CD86 expression were determined with respective Fluorescence Minus One (FMO) immunostainings. (**f, g**) Plots depict the frequency (median and 95% CI of the median) of CD86^+^ cells within membrane-bound CTLA-4^+^ (red) or CTLA-4^–^ (blue) mTeffs (**f**); or within HLA-DR^+^ (red) or HLA-DR^–^ (blue) mTeffs (**g**). Data was obtained following *in vitro* stimulation with αCD3/CD28 beads (1 bead: 3 cells ratio) in the presence (500U; solid line) or absence (dashed line – resting cells) of IL-2. Resting cells were defined as cells which were no longer re-stimulated with αCD3/CD28 after day 15.


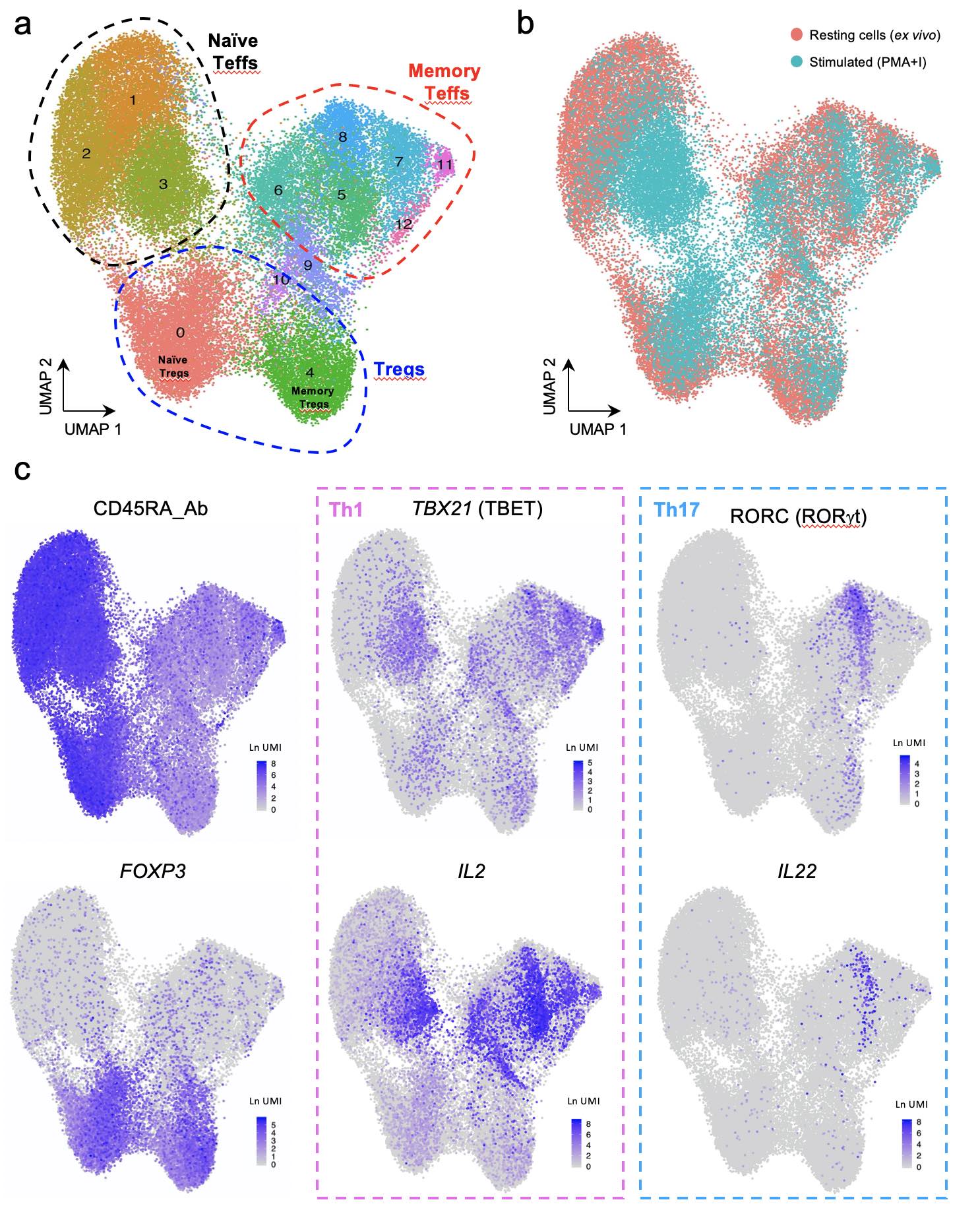


**Fig S8. Integration of data from resting and *in vitro* stimulated CD4^+^ T cells.**

(**a**) UMAP plot depicting clustering of all sorted CD4^+^ T cells captured in this study. Clustering was performed by integrating both resting and *in vitro* activated CD4^+^ T cells and corresponds to a total of 43,656 single-cells passing QC. Dashed lines correspond to the same manually annotated functional T-cell subsets depicted in Figs. 1-3. (**b**) Alignment of the integrated targeted transcriptomics and proteomics. Cells are colour-coded according to whether they originate from the resting (red) or *in vitro* stimulated (teal) datasets. (**c**) UMAP plots depicting the expression of representative markers used for the annotation of the functional T-cell subsets.


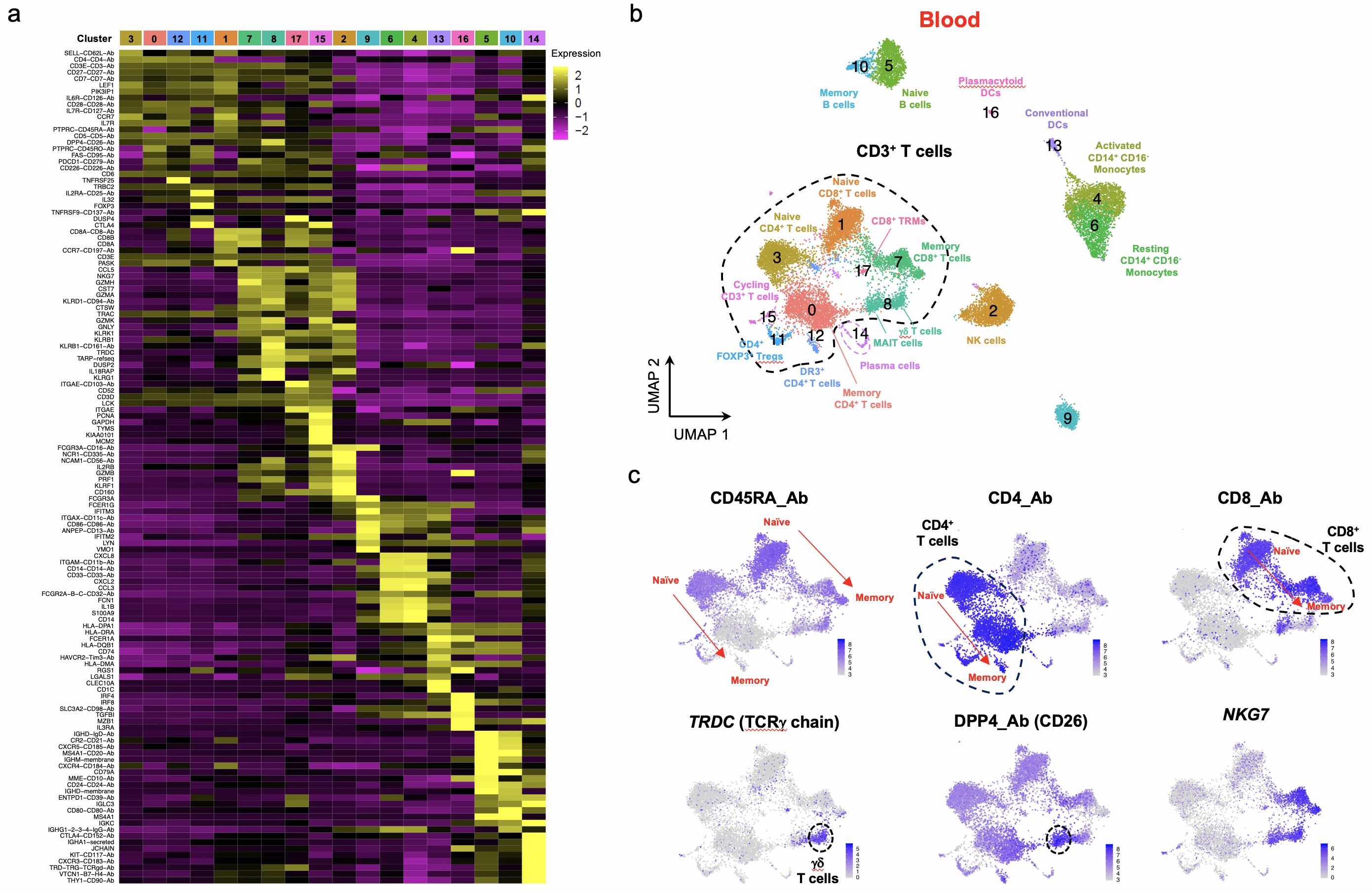


**Fig S9. Single-cell mRNA and protein quantification identifies distinct functional populations of human circulating CD3^+^ T cells.**

(**a**) Heatmap displaying the top 10 differentially expressed genes in each identified cluster from the CD45^+^ immune cells isolated from blood of two coeliac disease (CD) patients with active disease. (**b**) UMAP plot depicting the clustering of the circulating CD45^+^ immune cells. Dashed lines outline the annotated CD3^+^ T-cell clusters annotated from the differentially expressed genes in those clusters. (**c**) Functional annotation of the peripheral T-cell subsets using the expression profile of CD45RA and additional key lineage-defining T-cell markers such as CD4, CD8, *TRDC*, DPP4 and the effector cytokine gene *NKG7*. Arrows indicate the gradient of decreasing CD45RA and concomitant gain in CD45RO expression associated with the acquisition of a memory phenotype in response to antigen stimulation in CD4^+^ and CD8^+^ T-cells. DR3, death-receptor 3 (encoded by *TNFRSF25*); TRM, tissue-resident memory T cells; MAIT, mucosal-associated invariant T cells; DC, dendritic cells.


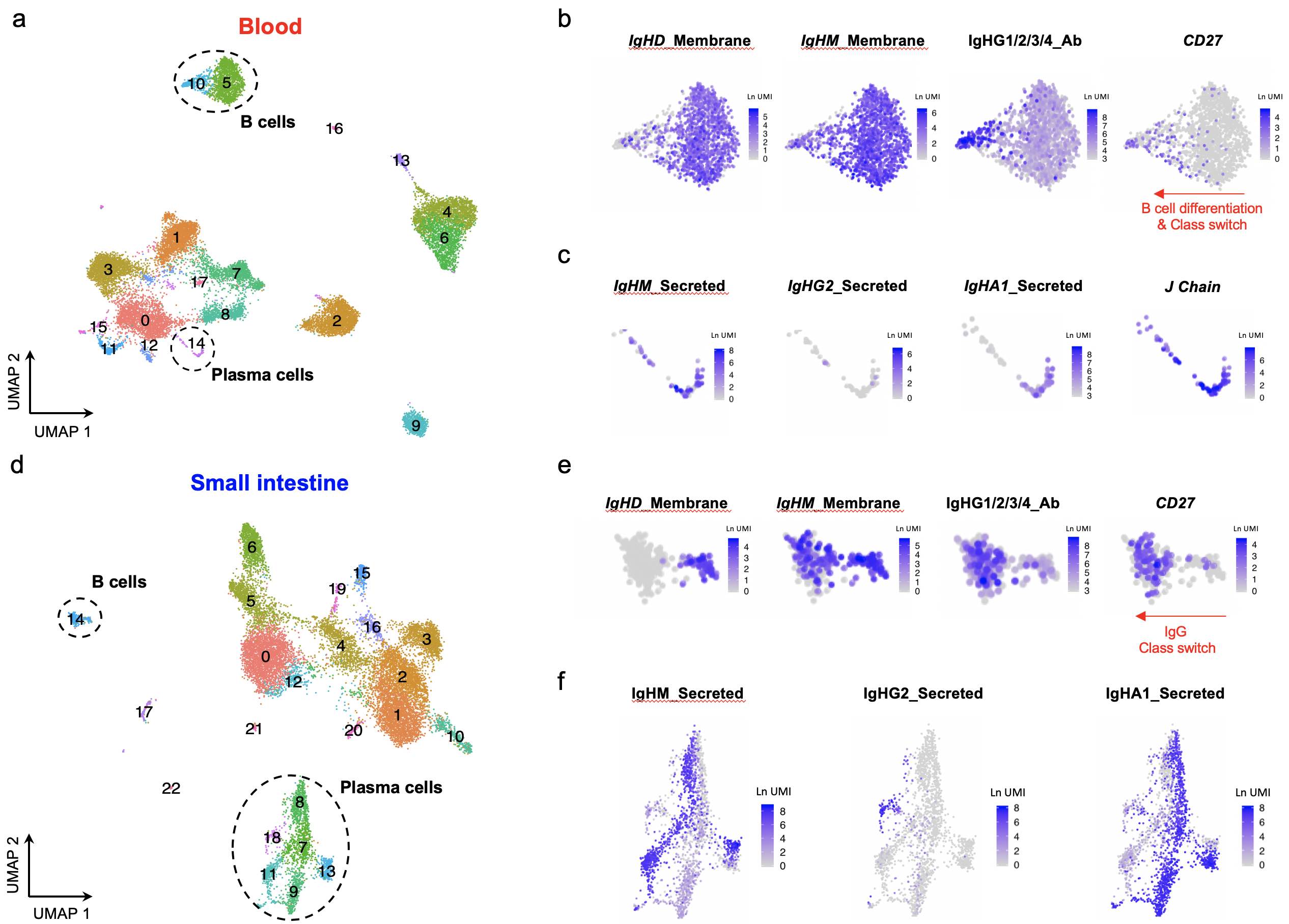


**Fig S10. Targeted multi-omics approach reveals trajectories of B-cell differentiation and class switching in blood and tissue.**

(**a**) UMAP plots depicting the clustering of the total CD45^+^ immune cells isolated from blood of two coeliac disease (CD) patients with active disease. Dashed lines outline the annotated B-cell and plasma cell subsets annotated from the differentially expressed genes in those clusters. (**b, c**) Expression of key B-cell differentiation and class switching (**b**) and plasma cell (**c**) markers, including *CD27* mRNA and selected surface expressed or secreted immunoglobulin (Ig) receptors in the identified circulating B-cell and plasma cell clusters. (**d**) UMAP plots depict the clustering of the total CD45^+^ immune cells isolated from duodenal tissue biopsies. Dashed lines outline the annotated B-cell and plasma cell subsets annotated from the differentially expressed genes in those clusters. (**e, f**) Expression of key B-cell differentiation and class switching (**e**) and plasma cell (**f**) markers, including *CD27* mRNA and selected surface expressed or secreted immunoglobulin (Ig) receptors in the identified in the tissue-resident B-cell and plasma cell clusters.
